# A principled representation of elongated structures using heatmaps

**DOI:** 10.1038/s41598-023-41221-2

**Published:** 2023-09-14

**Authors:** Florian Kordon, Michael Stiglmayr, Andreas Maier, Celia Martín Vicario, Tobias Pertlwieser, Holger Kunze

**Affiliations:** 1 Pattern Recognition Lab, Friedrich-Alexander Universität Erlangen-Nürnberg, 91058 Erlangen, Germany; 2 Erlangen Graduate School in Advanced Optical Technologies (SAOT), Friedrich-Alexander Universität Erlangen-Nürnberg, 91052 Erlangen, Germany; 3 Advanced Therapies, Siemens Healthcare GmbH, 91301 Forchheim, Germany; 4 Optimization Group, Institute of Mathematical Modelling, Analysis and Computational Mathematics, University of Wuppertal, 42119 Wuppertal, Germany

**Keywords:** Computer science, Computational science

## Abstract

The detection of elongated structures like lines or edges is an essential component in semantic image analysis. Classical approaches that rely on significant image gradients quickly reach their limits when the structure is context-dependent, amorphous, or not directly visible. This study introduces a principled mathematical description of elongated structures with various origins and shapes. Among others, it serves as an expressive operational description of target functions that can be well approximated by Convolutional Neural Networks. The nominal position of a curve and its positional uncertainty are encoded as a heatmap by convolving the curve distribution with a filter function. We propose a low-error approximation to the expensive numerical integration by evaluating a distance-dependent function, enabling a lightweight implementation with linear time complexity. We analyze the method’s numerical approximation error and behavior for different curve types and signal-to-noise levels. Application to surgical 2D and 3D data, semantic boundary detection, skeletonization, and other related tasks demonstrate the method’s versatility at low errors.

## Introduction

The detection of lines and contours is a central topic of image analysis and image understanding. Applications are widespread and range from computer vision, defect detection^1,2^, facial boundary and expression recognition^3–6^, geographic data analysis^7^ to medical imaging^8^. The images may be photographs of streets where the curbside should be detected or images of wheel rims where cracks are identified^9,10^. Other applications consider satellite imagery to monitor the extent of glacial termini^11,12^ or X-ray monitoring of a catheter during a surgical intervention^13^. While these applications differ in their nature, the underlying mathematical problem stays the same. A line, boundary, ridge, or contour—subsequently summarized by the term *elongated structure*—should be detected in an image.

Classical approaches typically tackle this task by using finite differences in intensity or color to approximate image gradients^14–17^. However, these methods fail when no common step representation can be found or if the noise level varies too much over the image. Active Contour Models^18^ alleviate this problem by minimizing the energy of a contour spline subject to some additional constraints. While a suitable energy term can be automatically derived by evaluating the contour-surrounding structures^19,20^, the fundamental problem of a good initialization remains, and these methods reach their limits when no shape model for the target structure can be found. This is especially the case for tasks in unconstrained settings like human pose estimation problems, where there is an infinite number of combinations of the person’s poses and clothes with the background^21,22^. For such problems, Convolutional Neural Networks (CNN) have become state of the art^23^. By sequential aggregation of features, abstract representation models can be optimized that generalize well across a large number of image impressions and shape realizations.

However, until now, no default methodology has been established for the representation of elongated structures and the subsequent formulation of an optimization target. This is particularly the case for structures that are not directly visible, do not have a specific shape or form, or are defined by their surrounding, often varying context. Current literature proposes several different strategies to address this detection task (Supplementary Material Table S1).

A typical approach is to formulate the optimization task as a *binary segmentation* problem, where the elongated structure is represented as a discretely sampled line of small width^24^. The algorithm is optimized to delineate the line pixels and all background using a (binary) cross-entropy (BCE) or soft dice (Dice) cost function. Unfortunately, segmentation inherently suffers from a substantial class imbalance if the target structure constitutes only a small foreground area. Also, label bias and minor offsets in the predicted segmentation strongly influence the optimization cost, rendering the optimization target volatile. As a result, the prediction is prone to fragmentation and gaps in ambiguous regions. One effective approach to mitigate this problem is a distance-weighted loss that relaxes the optimization target in the curve-near region^25,26^. Although this reduces fragmentation, it also smooths out potentially important true-positive gaps in the structure. Other approaches compose multiple segmentation cost functions^27^ or leverage regional integrals to penalize errors in contour space, effectively circumventing large magnitude differences present in overlap-based cost functions^28^.

A second approach is the extraction of the centerline/symmetry axis, often referred to as *skeletonization*. Traditional algorithms describe the centerline as the set of midpoints of maximally inscribed disks in an object^29^. The skeleton resulting from this definition is very sensitive to small boundary changes caused by noise or errors during boundary extraction^30^. Advanced algorithms alleviate these problems with costly post-processing^31^, define the task as a learning problem in scale-space^32^, utilize multi-task CNNs to combine skeleton pixel detection and skeleton scale regression^33,34^, or encode the center-line as points with positive inward flux in a locally confined vector field^35^. While the latter method does not presume explicit boundary information, flux areas of multiple objects in a scene must not overlap, which limits the application to arbitrary elongated structures.

Approaches for facial boundary detection mostly build upon the idea of *heatmap-based landmark localization* where the landmarks’ positional likelihood is encoded via spatial activation maps. These maps are typically computed by sampling a bivariate Gaussian function whose mean is at the ground truth coordinate^36,37^. By approximating a polyline that connects multiple structure-describing landmarks, this approach can be extended for elongated structures^22,23,38^. The polyline is mapped to a heatmap by either merging individual heatmap representations of the polyline points^3,39^ or by resolving pixel-wise distances to the structure using a distance transform^5,6,40,41^. However, an inherent issue with these approaches is the piecewise linear effect when connecting distant landmarks, causing inaccuracy at high-curvature regions which inversely scales with the number of landmarks^4^. Despite this effect, such representation is desirable as it provides high spatial generalization during inference and promotes a stable gradient signal for every spatial position, reducing the amount of data needed. Furthermore, the choice of the heatmap-generating function is theoretically arbitrary, making it easy to adapt the type of representation to the specific task.

Similarities are found in recent applications of graph convolutional neural networks (GCNNs) to line, contour, and semantics analysis^42,43^. The relations and constraints between salient positions of an elongated structure can be represented by the adjacency matrix of a graph. This allows us to incorporate global semantics and overcome topological inconsistencies which might appear upon image domain shifts or information loss by artifacts and occlusion. To this end, the typical process is to first locate relevant landmarks using image features and then perform semantic linkage-based graph analysis with GCNNs to obtain plausible landmark positions^42,44,45^.

**Figure 1:**
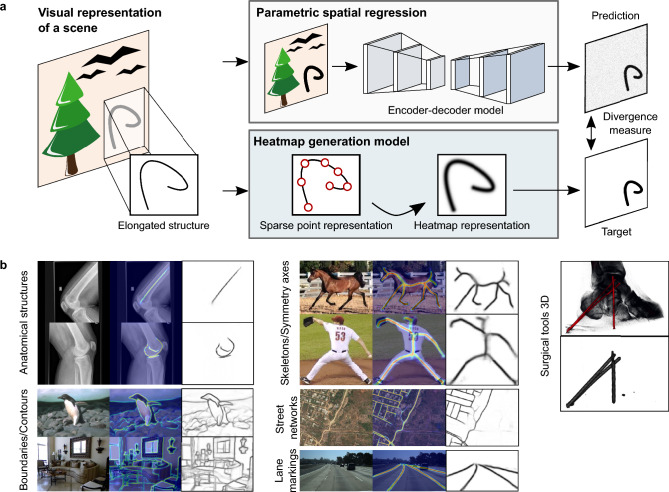
Conceptual overview and application examples. (**a**) Main use case of the heatmap representation as an operational description of the target function in a learning system. (**b**) application of the heatmap representation to anatomical feature detection, boundary detection^46,47^, skeletonization^33,48,49^, line detection^50^, segmentation^51^, and surgical tool detection in 3D (Sect. “Application to 2D and 3D representation problems”, Supplementary Material Sects. S3–S6).

In this work, we propose a mathematical description of elongated structures that offers a fundamental and holistic view of curves in space and provides a general encoding for a wide range of practical applications. This description shall serve, among others, as a target for optimization-based algorithms in which a neural network learns a representation of the elongated structures and approximates their distribution in space (Fig. 1). For these purposes, we adapt the concept of heatmap representation of single points to an arbitrary continuous curve in space with associated positional uncertainty. The distribution of curve points is convolved with a filter function, allowing prior domain knowledge of the expected signal and noise types to be included. To avoid numerical integration, we introduce a low-error approximation that simplifies the integral calculation to an evaluation of a distance-dependent function. To understand the representation’s fundamental properties, we analyze the numerical approximation error, compare the method to several related spatial representation types, study the influence of different signal/heatmap configurations, and evaluate the susceptibility to noise (Sect. “Analysis of representation properties”, Supplementary Material Sect. S2). The findings of this analysis are validated for various application examples in 2D and 3D (Fig. 1), coming with challenging and diverse types of elongated structures (Supplementary Material Table S2). For this purpose, the network is given a data sample as input and is tasked to approximate the structure’s position by matching the heatmap obtained using the proposed heatmap representation. This matching is supervised using a divergence measure, e.g., mean squared error, between the network prediction and the generated target.

We highlight our contributions to pattern analysis research in the following areas:

- We introduce a general mathematical description for 2D and 3D curves of various origins, shapes, and information levels. A primary application is the use of this description as an optimization target in CNN-based representation learning.
- We provide a numerical solution that is easily configurable and allows tailoring to the observed or expected curve type, the uncertainty of the curve’s position, and the application scenario. Unlike most conventional representation methods that rely on dense curve representations, this solution supports highly sparse curve signals with few sampled data points, completely avoiding the piecewise linear effect if desired.
- We study the numerical approximation error, showing negligible error magnitudes if no extreme curve curvatures exist. Furthermore, we draw clear lines of the proposed method to related spatial representations in the context of gradient-based optimization algorithms.
- We analyze the method’s properties for different curve types and representations. We show that Gaussian-distributed heatmaps are generally applicable for arbitrary signals, and derive implications for practical applications.
- We demonstrate the representation’s applicability as a target for CNN-based optimization for a broad array of tasks: anatomical feature detection (Sect. “Anatomical structure detection on knee radiographs”), 3D implant detection (Sect.  “Detection of surgical implants in 3D CBCT volumes”), object contour detection (Supplementary Material Sect. S3), skeletonization (Supplementary Material Sect. S4), road lane detection (Supplementary Material Sect. S5), and road network segmentation (Supplementary Material Sect. S6).

## Heatmaps for elongated structures

We start our considerations by looking at operational descriptions of target functions that a CNN-based learning system can adequately approximate. Fundamentally, the type and expressiveness of a representation ultimately define how well the target function, e.g., the distribution of a curve in space, can be approximated. They further dictate the number of data samples needed to train the learning system until it reaches a sufficient approximation fidelity^52^. On the other hand, the choice of representation, e.g., a spatial probability map or parametric encoding, influences which cost functions can be used. With these aspects in mind, we subsequently introduce a general and versatile mathematical description for elongated structures of different origins and shapes. We derive their representation as spatial heatmaps and propose a simple and fast implementation that allows for efficient network training.

### From convolution to distance function

The representation of an elongated structure as a heatmap can be motivated from several perspectives. On the one hand, heatmaps model the positional uncertainty of the curve. Thus, the position of a curve $\tilde{{\textbf{c}}}(t) = {\textbf{c}}(t) + {\textbf{z}}(t)$ with parameter variable $t\in [t_0,t_n]$ is the sum of the nominal position of the curve ${\textbf{c}}(t)$ superposed by a noise term ${\textbf{z}}(t)$ with probability density function $\textbf{pdf}$. By evaluating the parameter variable $t$ for the entire interval of definition from $t_0$ to $t_n$, it traces a noisy image of the curve in space. Moreover, a heatmap can be used as an optimization target for CNN training, where the correspondence with some ground truth is evaluated with a specific type of cost function. For the binary segmentation of an image into relevant curve points and background, BCE or Dice cost functions are used. If we can assume a normal distribution to model the curve point position with high likelihood, a least-squares-based cost is a natural choice.

Besides representing a curve explicitly as the image of a parameter-dependent function, it can also be described implicitly as a level set, which is defined by the (non-linear) equation

1 $$\begin{aligned} C = \left\{ {\varvec{x}} \in {\mathbb {R}}^n :f({\varvec{x}})=0 \right\} . \end{aligned}$$

The function $f({\varvec{x}})$ is a measure for the distance of a point ${\varvec{x}}$ to the curve. In this generic setting, it would suffice to use a function $f({\varvec{x}})$ which evaluates to $0$ at curve points. However, for simplicity of notation, we introduce $f$ as a distance measure here, which will be required in later sections. Using this distance function, the distribution ${\mathscr {D}}$ of a curve in the space ${\mathbb {R}}^n$ is

2 $$\begin{aligned} {\mathscr {D}}({\varvec{x}}) = \delta \left( f({\varvec{x}}) \right) , \end{aligned}$$

with $\delta (s),\,s\in {\mathbb {R}}$ being the Dirac function. We can now convolve this distribution with a filter function $w({\varvec{x}})$ and obtain the heatmap

3 $$\begin{aligned} H({\varvec{x}})&= {\mathscr {D}} ({\varvec{x}}) * w({\varvec{x}}) \nonumber \\&= \int _{{\mathbb {R}}^2} \delta (f({\varvec{y}})) \, w({\varvec{x}}-{\varvec{y}}) \,\textrm{d}{\varvec{y}} \nonumber \\&= \int _C w({\varvec{x}}-{\varvec{y}}) \,\textrm{d}{\varvec{y}} . \end{aligned}$$

However, solving $H({\varvec{x}})$ requires the evaluation of the line integral along a potentially curved line through the filter kernel. Depending on the implementation, no closed-form solution is available, so that numerical integration is necessary. For the special cases of rotationally symmetric functions and straight lines, this could be implemented using look-up tables. However, since we are interested in a general description that also includes curved lines, we introduce a simplification for the description of $H({\varvec{x}})$, which is considerably faster than numerical integration: In the following, we assume that $w$ is radially symmetric, i.e., $w({\varvec{x}})= w({\varvec{y}})$ for all ${\Vert {\varvec{x}}\Vert }_{2}$=${\Vert {\varvec{y}}\Vert }_{2}$. If we assume that $C$ is an infinite straight line, the evaluation of the integral simplifies to a function $h:{\mathbb {R}}\rightarrow {\mathbb {R}}$ which only depends on the distance to the line $C$:

4 $$\begin{aligned} H({\varvec{x}}) = h(f({\varvec{x}})). \end{aligned}$$

In the following, we will use (4) also in the general case as an approximation for the integral in (3).

**Figure 2:**
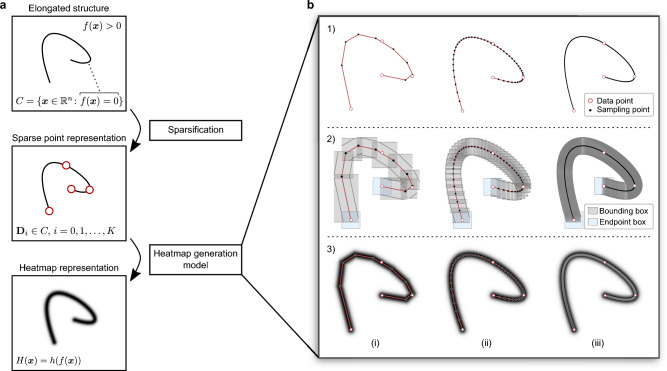
(**a**) General workflow of representing an elongated structure as a spatial heatmap. (**b**) heatmap generation model where (i)–(iii) mark 10, 50, and 500 curve sampling points, respectively. (**b1**) Calculation of 2D polylines which connect several sampling points from a parametric interpolation of the original curve signal. (**b2**) approximation of a compact hull that encloses the relevant spatial regions where point-to-curve distances are calculated. (**b3**) Heatmap representation of the curve using a Gaussian-distributed distance-dependent function.

### Numerical approximation and implementation

In this section, we describe an efficient implementation for this approximation of convolution by a distance-dependent function.

We assume that a finite set of data points can describe the original structure sufficiently well. These points are then used to derive a functional curve approximation by performing a (smooth) parametric B-spline interpolation. As only the spatial positions within a limited distance to the curve are numerically relevant for evaluating the distance function, we restrict calculations to a compact hull along the curve. Therefore, we sample the interpolating function and form a 2D polyline by connecting consecutive sample points (Fig. 2). For each resulting linear segment, a minimum enclosing rectangle (bounding box) is formed, and a distance value is calculated for each contained spatial position. After resolving multiple distance estimates obtained from overlapping hulls, the heatmap is calculated directly by evaluating the distance values with a distance-dependent function.

#### Curve parameterization

We assume that a curve $C\subset {\mathbb {R}}^n$ is described by a finite set of data points ${\textbf{D}}_{i}\in C ,\,i=0,1,\ldots ,K$ (cf. (1)). For non-trivial curves with $K > 1$, a smooth parametric B-spline is fitted to the original curve signal. The fitting criterion is based on the formulation by Dierckx^53^, where the trade-off between closeness and smoothness of fit is controlled with a smoothing factor $S$. There, the curve approximation $s({\textbf{D}}_{i})$ is optimized such that the sum of residual squares between the approximation and prescribed close interpolation $y({\textbf{D}}_{i})$ is equal to or smaller than $S$ so that $\sum _{i=0}^{K}(y({\textbf{D}}_{i}) - s({\textbf{D}}_{i}))^2 \le S$. Following Reisch^54^, $S$ is automatically derived from the number of data points with $S={|{\textbf{D}}_{i}|} - \sqrt{2{|{\textbf{D}}_{i}|}}$.This allows the approximating spline to be less sensitive to label or sampling noise. For curves with large local variance in curvature, S can be set to 0, returning an interpolating curve that picks up high frequencies more accurately. Every data point ${\textbf{D}}_{i}$ is assigned a parameter value $t_{i}\in [0,1]$. Evaluating $t_{i}$ over the interval of definition allows to smoothly trace the curve in space^55^. Since we do not make any assumptions on the curve type and its potential physical constraints but still want to keep the closeness of fit when tracing high-curvature regions (especially when the data points are not linearly distributed), we re-parameterize $t_{i}$ according to the *arc-length parameterization* method^55,56^. To this end, the arc-length parameters are approximated by dividing the domain of the interpolating curve using the chord-length ratios between ${\textbf{D}}_{i}$ and ${\textbf{D}}_{i-1}$^56–59^.

5 $$\begin{aligned} t_0 = 0, \quad t_i = \frac{1}{L_{h}} \,\sum \limits _{k=1}^{i}{\Vert {\textbf{D}}_{k} - {\textbf{D}}_{k-1}\Vert }, \quad t_n = 1 \end{aligned}$$

$L_{h}$ is the length of the polyline connecting the data points and corresponds to the sum of all chord lengths, i.e. $L=\sum _{i=1}^{K}{\Vert {\textbf{D}}_{i} - {\textbf{D}}_{i-1}\Vert }$. Floater et al.^58^ have shown that this approximation $L_{h}\approx L$ of the actual curve length $L$ is of second-order accuracy. The coord﻿inates $x_{i}$ and $y_{i}$ of the target B-spline control points ${\textbf{P}}_{i} \in {\mathbb {R}}^n,\,i=0,1,...,K$ can then be determined from the interpolation condition of the parametric spline

6 $$\begin{aligned} {\textbf{D}}_{i} = {\textbf{C}}(t_{i}) = \sum _{k=0}^{K} c_{k}\, B_{k,d}(t_{i})\,{\textbf{P}}_{k}, \end{aligned}$$

where $B_{k,d}(t_{i})$ are B-spline basis functions of degree $d$, and $c_{k}$ are the respective coefficients. This curve equation can be efficiently solved using Dierckx’s algorithm^53^. For $K>2$ control points, a cubic spline interpolation with $d=3$ is used, for $K=2$ the spline is of order $d=2$, and for a line segment with $K=1$ it simplifies to a linear interpolation, i.e. $d=1$. Once the interpolating function is found, $m$ points are sampled from the spline using a uniform sampling of $t_{i}$ at constant arc-length interval^55^. The connection of these curve points then yields a polyline of $m-1$ segments. Consequently, the number of segments is an adjustable parameter that regulates the quality of the approximation.

#### Local distance estimation

In the context of the proposed simplification, the distance evaluation for a single spatial position does not correlate with that of other points in ${\mathbb {R}}^n$. This enables a piecewise and localized evaluation of the heatmap for each polyline segment.

Following this idea, consider a narrow curve signal on a large background. This background, which can be noise or could provide any other information, has only a marginal contribution to the spatial configuration of this curve. When evaluating $f({\varvec{x}})$ and $h(f({\varvec{x}}))$, we are only interested in a small local hull around the curve for which we assume compact support. As we work with a polyline that consists of piecewise linear segments, we can translate the idea of a local hull by constructing the smallest enclosing box for each segment. In other words, given the start and end point of a single polyline segment, two parallel segments are set as margins at a distance of $+\,w/2$ and $-\,w/2$ defined by support $w$. Then, a minimum bounding box enclosing these two parallel lines is defined. When working with image datasets, bounding boxes are ideally axis-parallel to allow for efficient parallel computation. Since at both the start and end point of the polyline, the resulting hull does not fully extend into the direction of the curve, two additional boxes of width $w$ are centered on these points.

This approach allows the distance function $f({\varvec{x}})$ to be evaluated only for those points ${\varvec{x}}$, which are within one or more bounding boxes and therefore significantly contribute to the heatmap. Within each bounding box, the point-to-line distance is calculated for every contained spatial location ${\varvec{x}}$. For a line segment with start point $l_{1}$ and end point $l_{2}$, this distance equates to $f({\varvec{x}}) = {\left\Vert {\varvec{x}}-\Bigl ( l_{1}+s \cdot \frac{b}{{\Vert b \Vert }}\Bigr )\right\Vert }$ with $a = {\varvec{x}}-l_{1}$, $b = l_{2}-l_{1}$, and $s = \max \left\{ 0,\; \min \Bigl (\frac{a \cdot b}{{\Vert b \Vert }}, {\Vert b \Vert }\Bigr )\right\}$. Since bounding boxes will overlap for non-zero curvatures $\kappa >0$ or if the curve is self-intersecting, we will likely obtain multiple distance estimates $\textbf{f}_{{\varvec{x}}}=\left( f_{1}({\varvec{x}}), f_{2}({\varvec{x}}),\ldots , f_{n}({\varvec{x}})\right) \in {\mathbb {R}}^2$ for some subset of the hull points. This number of individual estimates, i.e., ${\Vert \textbf{f}_{{\varvec{x}}}\Vert }$, also strongly depends on the number of sample points used to construct the polyline and increases for higher sampling frequencies as the ratio between the support $w$ and the distance between sample points gets larger.

In order to map $\textbf{f}_{{\varvec{x}}}$ to a single distance scalar that can be evaluated with $h(\cdot )$, two options for a reduction function $h^{*}(\textbf{f}_{{\varvec{x}}})$ are proposed.

##### Minimum distance

The most simple reduction follows a *winner-take-all* approach where only the estimator with the smallest point-to-line distance is used for evaluating the distance-dependent function $h(\cdot )$.

7 $$\begin{aligned} h^{*}(\textbf{f}_{{\varvec{x}}})=h\bigl ( \min \{ f_i({\varvec{x}}) :i\in \{1,\ldots ,n \}\}\bigr ) \end{aligned}$$

This calculation can be easily parallelized, allowing the heatmap generation algorithm to be executed at linear time complexity $f \in {\mathscr {O}}(N)$, depending on the total number of spatial positions evaluated in the bounding boxes $N$ (c.f., Supplementary Material Fig. S8).

##### Inverse distance weighting

An inverse distance weighting (IDW) following Shepard^60^ allows the contribution of all distance estimates. IDW assigns an influence weight to each estimate that directly depends on the point-to-line distance.

8 $$\begin{aligned} h^{*}(\textbf{f}_{{\varvec{x}}})={\frac{\langle \lambda _{{\varvec{x}}}, h(f_i({\varvec{x}}))\rangle }{{\Vert \lambda _{{\varvec{x}}}\Vert }_{1}}}, \quad \text {where}\;\lambda _{{\varvec{x}},i}={\frac{1}{f_i({\varvec{x}})^{p}}} \end{aligned}$$

The power parameter $p$ controls each estimator’s relative strength and is set to $p=1$ (linear) or $p=2$ (quadratic).

#### Selection of the distance-dependent function

In principle, any 1D function can be chosen as $h(\cdot )$. However, since the resulting heatmaps are supposed to approximate the spatial position of a curve with some superimposed noise, the $\textbf{pdf}$ of a parametric probability function is a natural choice. In the following, we consider various zero-mean $\textbf{pdf}$ variants that are either frequently observed signal/noise shapes (Gaussian, Laplace, cosine) or are versatile in their encoding characteristics w.r.t. learning-based algorithms (triangular, rectangular) (c.f. visual representation in Supplementary Material Fig. S9). We also consider a custom signal shape in the form of a simulated hollow-core catheter profile. All distributions are defined with the assumption of compact support $\pm 3\sigma$.

*Gaussian*:9*1D Laplace/2D Super-Gaussian*:

10 $$\begin{aligned} h_{\text {slp}, \sigma }(f({\varvec{x}})) = \eta \, \exp {\left( -\frac{{|f({\varvec{x}})|}}{\sigma } \right) }, \quad \eta =\frac{1}{2\sigma } \end{aligned}$$

*Triangular*:

11 $$\begin{aligned} h_{\text {tri}, \sigma }(f({\varvec{x}})) = \eta \, \max \left( \frac{3\sigma - {|f({\varvec{x}})|}}{3\sigma }, 0\right) , \quad \eta =\frac{1}{3\sigma } \end{aligned}$$

*Rectangular*:

12 $$\begin{aligned} h_{\text {rec}, \sigma }(f({\varvec{x}})) = \eta \, {\left\{ \begin{array}{ll} 1 &{}{|f({\varvec{x}})|} \le 3\sigma \\ 0 &{}\text {otherwise} \end{array}\right. }, \quad \eta =\frac{1}{6\sigma } \end{aligned}$$

*Raised Cosine*:

13 $$\begin{aligned} h_{\text {cos}, \sigma }(f({\varvec{x}}))&= \eta \,{\left\{ \begin{array}{ll} \frac{1}{2} \bigl (1+\cos \bigl ({\pi \frac{{|f({\varvec{x}})|}}{3\sigma }}\bigr )\bigr ) &{} {|f({\varvec{x}})|} \le 3\sigma \\ 0 &{} \text {otherwise}\end{array}\right. }\nonumber \\ \eta&=\frac{1}{3\sigma } \end{aligned}$$

*Simulated catheter profile*:

14 $$\begin{aligned} r_{o}&= 3\sigma ,~r_{i} = s r_{o},\quad s\in [0,1] \nonumber \\ y(r)&= \sqrt{\max \bigl \{r^{2}-{|f({\varvec{x}})|}^{2}, 0\bigr \}} \nonumber \\ h_{\text {cat}, \sigma , \text {s}}(f({\varvec{x}}))&= \eta (y(r_{o}) - y(r_{i})) \nonumber \\ \eta&=\frac{2}{\pi (r_{o}^{2}-r_{i}^{2})} \end{aligned}$$

$r_{o}$ and $r_{i}$ mark the radius of an outer and inner circle corresponding to a catheter’s outer and inner wall. By changing the scaling parameter $s$ to shrink or expand the inner circle, different wall thicknesses of the tube can be simulated. In general﻿, when interpreting the heatmap as such a $\textbf{pdf}$ (which excludes the catheter signal), it is defined to be non-negative and to have a unit integral over its domain. Since we want to use the heatmap in gradient-based learning algorithms in which normalization of the target image is common, we propose to incorporate this normalization in the heatmap calculation such that $\max _{{\varvec{x}}\in {\mathbb {R}}^{n}} {h({\varvec{x}})}=1$. We argue that this allows for a more stable gradient across different heatmap parameterizations and reduces the risk of vanishing gradients due to small loss magnitudes. As a result, the normalization constant in (9)–(13) is set to $\eta =1$, whereas in (14) it is set to $\eta =\sqrt{r_{o}^{2}-r_{i}^{2}}$.

#### Extension to 3D

The localized calculation of relevant distance estimates is easily extendable to 3D. After finding a three-dimensional function to interpolate between a set of data points and extracting a corresponding polyline, the local hull is approximated by extending the two-dimensional bounding boxes to right rectangular prisms. A single prism is formed by calculating four equidistant line segments that are parallel to the polyline segment. A prism width of $w$ allows to obtain the minimum-area enclosing cuboid of the hull that is axis-aligned w.r.t. a Cartesian coordinate system (Supplementary Material Fig. S10).

## Analysis of representation properties

In this section, we analyze the method’s general numerical approximation error, its behavior for different shape and width configurations, and the relation between sampling ratio and curvature (c.f. Supplementary Material Sect. S2 for analysis of different signal-to-noise ratios). Furthermore, we compare the method’s approximation error with that of several related spatial curve representation and approximation methods.

### Approximation error for different curvature, heatmap widths, and reduction functions

As shown in Sect. “Heatmaps for elongated structures”, the approximation yields exact values for straight lines. However, this is no longer true for curved lines. In the following, we analyze the error introduced by this approximation with distance-dependent weights. We compare the approximation with a numerically evaluated convolution of the curve with the **pdf** of a normally distributed signal.

For this purpose, we devised an experimental setup with a simulated plane curve. By altering the curve’s spatial configuration, we can analyze the approximation error for various curvatures, widths, and reduction functions.

#### Experimental setup to measure the signal representation error

A plane curve $C$ parameterized by three data points $\{{\textbf{D}}_{0}, {\textbf{D}}_{1}, {\textbf{D}}_{2}\}$ is placed on a background of $\text {H:}2000 \times \text {W:}2000\,\textrm{px}$ following a spatial configuration of ${\textbf{D}}_{0} = (1500, 500)$, ${\textbf{D}}_{1}=(500, [500 + \delta \,1000])$ and ${\textbf{D}}_{2}=([1500 - \delta \,1000], 1500)$ (Supplementary Material Fig. S11). The hyper-parameter $\delta$ controls the angle between the two curve sections ($[{\textbf{D}}_{1}, {\textbf{D}}_{0}]$ and $[{\textbf{D}}_{0}, {\textbf{D}}_{2}]$) and allows to successively increase the maximum curvature of the curve observed at ${\textbf{D}}_{0}$. For every configuration $\delta \in \{0, 0.1, 0.2, ..., 1\}$, we compute the absolute error $\varepsilon$ that accumulates between the definite surface integral of curve $C$ and its numerical approximation at various parameterizations (c.f. Supplementary Material Fig. S11 and Supplementary Material Table S7):

15 $$\begin{aligned} \varepsilon = \int _{-3\sigma }^{3\sigma }{\left|\int _{{\textbf{S}}_{i}}{H({\varvec{s}}) \,\textrm{d} {\varvec{s}}} - \sum _{{\varvec{e}} \in {\textbf{E}}_{i}}{l_{{\varvec{e}}} h^{*}(f({\varvec{e}})) }\right|} \,\textrm{d} i. \end{aligned}$$

${\textbf{S}}_{i}\subset {{\mathbb {R}}^2}$ denotes an infinite set of points at an orthogonal distance $f(x)={|i |}$ to $C$ while respecting the orientation indicated by the sign of $i$. Consequently, ${\textbf{S}}_{0}=C$ (cf. (1)). ${\textbf{E}}_{i}\subset {{\textbf{S}}_{i}}$ is a finite subset of points that act as evaluation points for the approximated heatmap. The evaluation points ${\textbf{E}}_{0}$, which are located directly on the curve, are chosen by performing a uniform sampling of the curve parameter $t$. $l_{{\varvec{e}}}$ marks the individual chord-length between consecutive points in ${\textbf{E}}_{0}$. All other evaluation points sets ${\textbf{E}}_{i}$ with $i\ne 0$ are sampled such that they are located on the orthogonal line to the curve intersecting the corresponding point in ${\textbf{E}}_{0}$. By assuming a large enough number of evaluation points, we ignore that the distance between the evaluation points is not equally spaced for $f(x)\ne 0$ in the case of non-trivial curves that do not follow a straight line.

We can now obtain a normalized error ${\hat{\varepsilon }}=\frac{1}{L}\, \varepsilon$ by division with the total curve length $L$. A normalization across different parameterizations of the support of $h(\cdot )$ is not necessary since we normalize $\int {h(f({\varvec{x}})) \,\textrm{d} {\varvec{x}} = 1}$ for all $\sigma$ upon calculating the error values.

**Figure 3:**
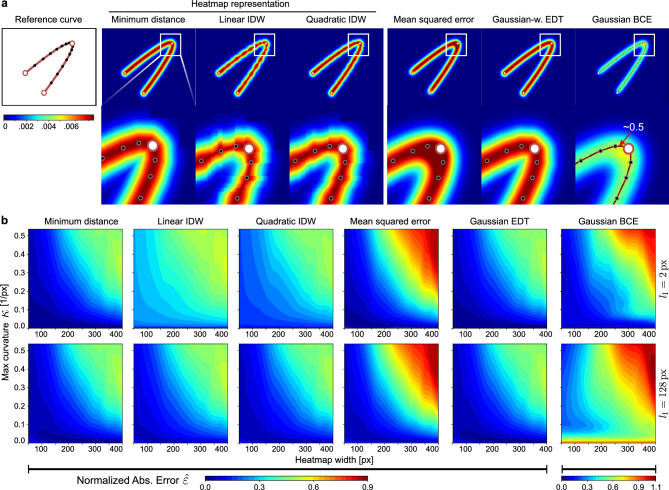
Analysis of approximation errors. (**a**) Comparison of spatial curve representations constructed according to Supplementary Material Table S7 with $\delta =0.8$, a heatmap width given by $\sigma =68\,\textrm{px}$, and a mean interpolation point distance of $l_{\text {I}}=128\,\textrm{px}$. (**b**) Error contour maps w.r.t. maximum curvature $\kappa$ and different heatmap widths $\sigma$. The color levels encode the normalized absolute error ${\hat{\varepsilon }}$ between the approximation and the theoretical integral along the curve according to (15).

#### The influence of the reduction function ${h^{*}(\cdot )}$

In the case of a densely sampled polyline (Fig. 3), both IDW schemes show a substantial noise floor. This can be explained by the presence of many overlapping bounding boxes, which leads to multiple distance estimates for most spatial locations within the hull. This implies an increase in large distance values, which causes a general underestimation of the heatmap values, especially for a linear IDW. Interestingly, this underestimation produces a smooth grid structure for both IDW variants, which particularly emerges in areas of high signal curvature and can be explained by a disproportionate accumulation of large distance estimates originating from oppositely located curve segments. Using the minimum distance circumvents this issue as only the closest estimate is considered, effectively filtering out most estimation noise. Sparse sampling of the curve reduces the error difference between the different reduction functions (Fig. 3), although at the cost of overall precision.

#### The influence of the curve/heatmap width and curvature

**Figure 4:**
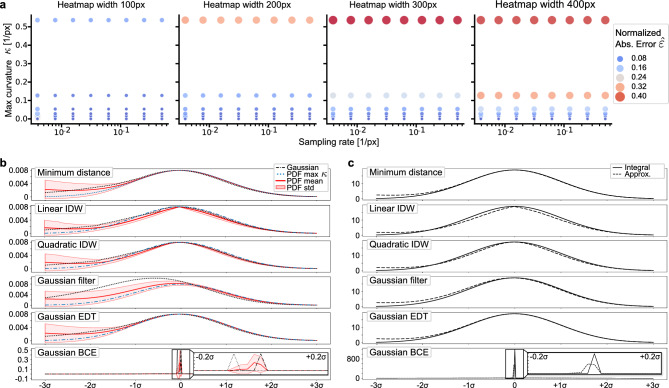
(**a**) Analysis of the relationship between maximum curvature ($\kappa$) and sampling rate ($1/l_{\text {I}}$). The size and color of the measurements mark the normalized error ${\hat{\varepsilon }}$ of the curve’s heatmap representation according to (15). (**b**) Curve profiles of the optimal Gaussian **pdf** and the profiles for max curvature and mean/std statistics. (**c**) Accumulation of the approximate and the theoretical integral along the curve ($l_{\text {I}}=128\,\textrm{px}$, $\delta =0.6$).

Across all reduction functions, a large curve/heatmap width increases the susceptibility to errors in high curvature sections. This results in error peaks of $\sim 60\,\mathrm {\%}$ of the accumulated signal for maximum curvatures in the range of $\kappa \in [0.25, 0.50]\,[1/\text {px}]$ and widths above ($w\ge 200\,\textrm{px}/\sigma \ge 34\,\textrm{px}$), which corresponds to 7% and 1.2% of the image diagonal, respectively. Such a value combination implies that the curve at least partly intersects with itself, which is neither considered in the three reduction functions nor the practical implementation (Sect. “Numerical approximation and implementation”). As the heatmap width increases, this overlap area expands, causing a general overestimation of the heatmap values due to high-valued distance estimates (Fig. 4).

#### The relation of sampling rate and curvature

To relate curvature and sampling rate using the Shannon-Nyquist theorem, we consider their relationship in the context of a curve’s highest frequency component. When a curve has high curvature, it undergoes rapid changes in direction, which corresponds to higher frequency components in the curve. The maximum curvature ($\kappa$) is given as the reciprocal of the radius of curvature and represents the curve’s maximum bending. The maximum curvature can be related to the highest frequency component through $f_{max} = \kappa / (2\pi )$. Now, the sampling theorem mandates a sampling rate at least double the highest frequency component to avoid aliasing, leading to the inequality $r>4\pi \kappa$ for the sampling rate $r$. This relationship guides the selection of an appropriate sampling rate during sparse sampling of the curve, ensuring accurate capture of the curve’s curvature and avoidance of aliasing effects.

At the same time, recall that we explicitly model the spatial uncertainty of the curve’s position, resulting in non-zero widths in the heatmap representation. The sampling quality thus also strongly depends on the chosen heatmap width. This is shown in Fig. 4, where the approximation error increases for larger curvatures across all sampling rates (reciprocal of sampling point distance $1/l_{\text {I}}$). Although there are small improvements in approximation quality from higher sampling rates, these must be weighed against the higher computational requirements. Moreover, since the width of the heatmap shows the highest impact on approximation fidelity, we argue that a self-overlapping of the curve or its noise spread is the main reason for larger errors in the approximation. Consequently, high curvatures can be most effectively tackled by choosing a small-to-medium-sized heatmap width (if possible). In the practical setting, signals with maximum curvature of up to $\kappa \approx 0.1 [1/\text {px}]$ can be modeled with negligible errors with little requirements w.r.t. the sampling rate.

### Comparison to related curve representations

Using the same experimental setup as in Sect. “Approximation error for different curvature, heatmap widths, and reduction functions”, we compare the method’s error to that of several related spatial curve representations. These share similarities in that they evaluate the point-to-curve distance, use a Gaussian model to assess the positional likelihood of the curve points or are rooted in a probabilistic interpretation of conventional cost functions in optimization algorithms (Fig. 3).

#### Mean squared error on curve points

We construct a spatial representation of the curve by filtering the discretized curve points with a Gaussian low-pass filter. This rationale can be interpreted as an evaluation of the mean squared error of the individual curve points, where the squares of the errors correspond to the exponent in the **pdf** of a Gaussian distribution and a constant normalization offset. The approximation errors obtained by this representation under various configurations are illustrated in Fig. 3. Since the number of convolution operations per spatial position increases sharply in the near-curve region with high curvature, we see high approximation errors with increasing signal complexity. This aliasing leads to a systematic broadening and migration of the distribution peak towards the inside of the curve, which reduces the likelihood of the curve points given the Gaussian model (Fig. 4).

#### Gaussian-weighted Euclidean distance transform

Discretizing the curve points lets us derive a representation of the curve as a distance field by means of Euclidean distance transform (EDT)^61^. Each position in this field corresponds to the Euclidean distance to the nearest discrete curve point (Fig. 3). Following (4), these distances can be elegantly evaluated with a Gaussian **pdf**. The observed approximation errors are almost identical to the proposed numerical approximation, and the smoothness of the curve similarly increases with the number of sampling points. However, for configurations with lower spatial resolution and more compact support of the **pdf**, the curve discretization and the resulting binning of distance estimates introduce stair-step artifacts (Supplementary Material Fig. S8) that could be detrimental in some scenarios. However, for extremely large noise spread and very dense curve sampling, this representation might be preferred due to very efficient computation with linear time complexity $f \in {\mathscr {O}}(N)$^61^ w.r.t. the number of image pixels $N$ with no dependency to the number of sampling points and $\sigma$. In such a scenario, no time can be saved by evaluating only the numerically relevant distances in the regions near the curves (as in the proposed representation) due to excessive bounding box overlap (Supplementary Material Fig. S8).

#### Gaussian-weighted BCE

We implement the rationale of distance weighting directly in a BCE loss function, which is typically used for optimizing binary segmentation problems. For that purpose, a discrete 2D convolution of the curve points and the impulse response of the BCE term is calculated. Each position in the impulse response is weighted using a Gaussian **pdf** (Fig. 3). The approximation error is calculated by comparing the resulting curve profile with the corresponding 1D impulse response. Similar to the MSE variant, we observe peak flattening and migration towards the inside of the curve, which is increased in high curvature areas with large noise spread. Interestingly, sparse sampling of the interpolation function results in larger errors in regions with little curvature. Due to the impulse response’s extremely narrow and high-magnitude peak, even minor offsets between the original curve and the approximation lead to large errors. This behavior is a direct result of the B-spline interpolation scheme, where the relative amount of low-curvature sections in the curve legs increases with a higher curvature at the turning point.

### CNN-based estimation error for different signal and heatmap configurations

A primary application of the presented representation is its use as an operational description of the target function in a CNN-based learning system. This raises the question of how the configuration of the observed type of target signal that should be approximated responds to different the configuration of the heatmap. To address this question, we use a signal simulation model to construct pairings of varying signal and heatmap configurations and compare the predictive performance of a reference CNN (Sect. “Simulated signals on chest X-ray”, Supplementary Material Table S8) using the Average Symmetric Surface Distance (ASSD) metric (Sect. “Variant 1: ASSD”). Here, we take advantage of the fact that our method not only allows us to construct heatmaps that approximate a specific signal but also enables the simulation of an arbitrary elongated structure itself. To generate non-trivial and realistic input images for the neural network, the simulated structures are superimposed on the human chest radiographs (Sect. “Simulated signals on chest X-ray”).

**Figure 5:**
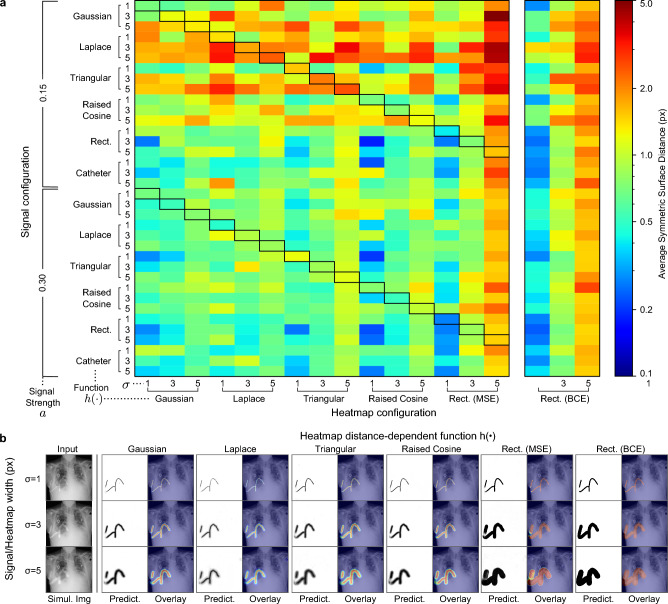
CNN estimation error for various signal/heatmap configurations. (**a**) Heatmap of mean test ASSD for signal/heatmap configurations. $a$: signal overlay strength, $\sigma _{\text {sim}}/\sigma _{\text {hm}}$: signal/heatmap width as standard deviation (px), $h(\cdot )$: distance-dependent function. Black boxes mark matching signal and heatmap configurations. (**b**) CNN predictions for Gaussian-distributed signals. The test sample was selected according to the smallest difference to the mean ASSD over all signals with $a=0.15$.

#### The relation between a signal distribution and heatmap distance-dependent function

We observe a weak correlation between the signal distribution and the distance-dependent function of the heatmap (Fig. 5). Only using a rectangular distribution leads to a substantially worse reconstruction of the original curve, with the ASSD metric reaching error values of up to $5\,\textrm{px}$. Mesokurtic choices of the distance-dependent function (Gaussian, Raised Cosine) generally work well for all types of signal configurations. The custom catheter signal (Sect. “Selection of the distance-dependent function”) is estimated with negligible error for all heatmap configurations. Similar to the rectangular distributed signal, the signal strength plays a minor role due to high intensities across the discrete support.

#### The influence of signal and heatmap width

A perfect match between the signal and heatmap width does not benefit the representation quality during network inference. On the contrary, a small heatmap width is generally favorable regardless of the signal configuration. This observation is of great advantage in real-life applications where we cannot assume a fixed signal width or where little information of the signal configuration is available. As shown in Fig. 5, the rectangular distribution shows the largest error with increasing heatmap widths. Due to its uniform intensity profile, a high width forces the network to significantly overestimate the signal intensity distribution, especially for leptokurtic signals (Laplace, Triangular). This representation bias is slightly reduced upon the optimization with BCE but does not change the general error tendency.

#### The limitations by signal strength

For all but a rectangular signal shape, the strength of the signal overlay and, consequently, the separability between background and signal based on image information plays a dominant role. For weaker signals with $a=0.15$, the predicted heatmaps suffer from increased fragmentation, frayed edges, and underestimated intensity values. Especially for wide and leptokurtic signals, such little contrast significantly impedes the reconstruction quality of the original signal curve. This behavior is expected since small deviations of the center-line during network inference can lead to much stronger mass shifts than what can be observed for mesokurtic or platokurtic signals, directly affecting the determined Otsu threshold and skeletonization.

## Application to 2D and 3D representation problems

In the following section, we evaluate the applicability of our approach to real-life application examples. Complementing evaluation on other datasets and curve types is provided in Supplementary Material Sects. S3–S6.

### Anatomical structure detection on knee radiographs

We evaluate our method for the detection of anatomical structures and a surgical reference line on 2D radiographs (Sect. “Datasets and training protocols”). We analyze heatmap representations of four elongated anatomical features that are part of the human knee joint, ranging from edges/boundaries, curved and straight lines partially supported by image information, and a contextual center-line^62,63^ (Supplementary Material Fig. S12): (A) Blumensaat line in the intercondylar notch of the distal femur (curved). (B) Mean contour of the medial and lateral femoral condyles (curved). (C) Plateau line of the proximal tibia (straight). (D) Anatomical axis of the femur bone (straight).

Features (A)-(C) are structures that can be *directly* delineated on lateral radiographs by perceivable contrast in the respective areas or by semantically connecting salient landmarks. The anatomical axis (D) is not directly derived from image information but instead relies on the global orientation of the bone given by the surrounding bone shaft contours. We are particularly interested in the learning algorithm’s capability to infer such *contextual* structures. The directly perceivable features were trained jointly using a single model, and the contextual axis feature was trained individually.

**Figure 6:**
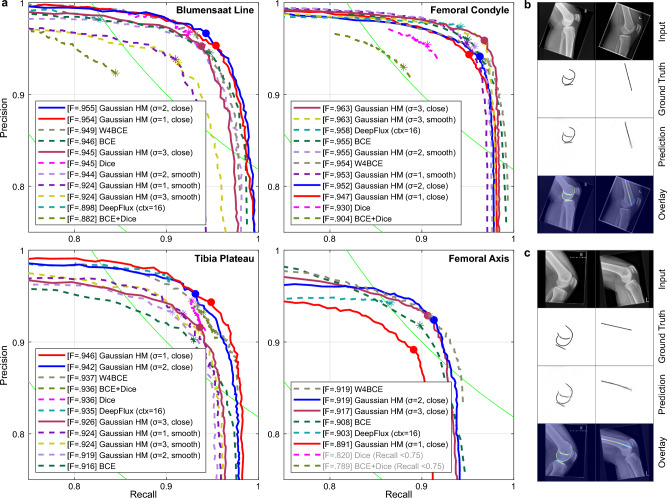
Performance analysis for representation of diverse anatomical features. (**a**) PR curves with F-measure (ODS). (**b**) Low-error predictions. (**c**) Erroneous samples.

As shown in Fig. 6, Table 1, and Supplementary Material Fig. S13, the heatmap predictions for both *direct* and *contextual* anatomical structures are of overall high spatial precision. The F-measure for the contextual femoral axis (D) is slightly lower than the corresponding scores for direct features (A)-(C) which are supported by salient anatomy. The observed outliers with low individual F-measure scores are caused by anatomical deviations, slight misalignment of the femoral condyles, or joint deformities such as ventral/dorsal bending of the bone. For this task, we can derive a relation between the optimal heatmap width $\sigma _{\text {HM}}$ and the type of structure to be represented. While we observe better detection rates for delicate structures like the Blumensaat line and plateau line for smaller widths, more pronounced and larger structures like the femoral condyle and axis benefit from a larger width. Furthermore, a close interpolation is preferable, which is particularly evident for intricate structures with detailed curves like the Blumensaat line. This difference, however, is negligible for larger structures like the femoral condyle. Interestingly, although the tibial plateau is a straight line and its interpolation doesn’t change upon different smoothing conditions, its detection performance benefits from using a close interpolation for the other two features in the combined training. In comparison, the segmentation and skeletonization methods achieve high but inferior F-measure scores compared to the heatmap representation. In particular, optimization with a Dice loss or the combination of Dice and BCE is significantly worse, achieving comparatively low recall for the femur axis.

**Table 1:** F-measure (ODS) comparison of different methods to detect anatomical structures on knee joint radiographs.

Method	Blumensaat line	Femoral condyle	Tibia plateau	Femur axis
BCE	0.946	0.956	0.916	0.908
Dice	0.945	0.930	0.936	0.820
BCE+Dice${}^{1}$	0.882	0.904	0.936	0.789
W4BCE${}^{2}$	0.949	0.954	0.937	**0.919**
DeepFlux-P${}^{3}$	0.898	0.958	0.935	0.903
Gaussian heatmap				
$\sigma _{\text {HM}}=1$ (smooth)	0.924	0.953	0.924	N/A
$\sigma _{\text {HM}}=2$ (smooth)	0.944	0.955	0.919	N/A
$\sigma _{\text {HM}}=3$ (smooth)	0.924	**0.963**	0.924	N/A
$\sigma _{\text {HM}}=1$ (close)	0.954	0.947	**0.946**	0.891
$\sigma _{\text {HM}}=2$ (close)	**0.955**	0.952	0.942	**0.919**
$\sigma _{\text {HM}}=3$ (close)	0.945	**0.963**	0.926	0.917

Corresponding analysis with the ASSD metric is provided in Supplementary Material Table S9. Comparison methods are (1) BCE+Dice^27^, (2) BCE + Dice^26^, (3) DeepFlux-P^35^ (ctx = 16).

Best value for each anatomical structure is in bold.

### Detection of surgical implants in 3D CBCT volumes

Surgical treatment of fractures or joint deformities is often guided by 3D cone-beam computed tomography (CBCT). Within these volumes, knowing the position and orientation of metallic implants like screws and k-wires is crucial to ensure the effectiveness and safety of the intervention. Using this task as an example, we investigate the use of 3D heatmaps and their applicability for representing elongated 3D structures (Sect. “Datasets and training protocols”). For evaluation and reporting, we use previously described signal extraction and ASSD metric (Sect. “Signal extraction and evaluation metric”), which can be easily extended for 3D curves.

**Figure 7:**
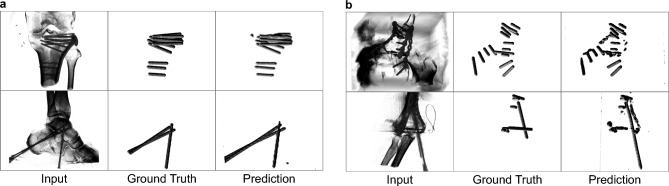
Analysis of detection performance of surgical screws and k-wires on 3D CBCT volumes. (**a**) Low-error predictions. (**b**) Erroneous samples.

Both screws and k-wires are reliably detected with high spatial precision (Fig. 7). Even in complex cases where a substantial number of implants are close to each other, the orientation and shape of each object instance are inferred with reasonable accuracy. However, in direct comparison to the 2D examples, the prediction quality is slightly lower. For all test data with metal artifact reduction (MAR) (Sect. “Datasets and training protocols”) and a Gaussian reconstruction filter size of $\sigma _{\text {rec}}=1$, a median ASSD of $4.59\,\textrm{voxels},~\text {CI}_{95\%}[3.65, 7.43]$ is achieved. Qualitative analysis of the high-error cases reveals that false-positive response at metallic plates, a heatmap intersection of close neighboring objects, and over-/underestimation of object length are the main reasons for failure. The latter can be attributed partly to ambiguous ground truth in anatomically complex and cluttered regions. Interestingly, using the same filter size but no MAR method yields a comparable score of $5.36\,\textrm{voxels},~\text {CI}_{95\%}[3.79, 6.66]$. This suggests that the proposed 3D heatmap representation can successfully overcome a reduction in image quality and that meaningful heatmap extrapolation in compromised areas is possible.

## Discussion and conclusion

This work introduces and investigates a general and versatile mathematical description for curves in space of various origins, shapes, and information levels. The primary purpose of this representation is to be used as a target in optimization-based algorithms that should learn an encoding of the structures and approximate their location in space. Given the distribution of a curve in space and an estimate of its positional uncertainty, a heatmap is generated by convolving this distribution with a filter function. Instead of a time-consuming numerical integration, we approximate the convolution by evaluating a freely selectable distance-dependent function. Since this function depends exclusively on a single spatial position, this simplification allows a localized, piece-wise, and thus easily parallelizable evaluation of the heatmap-generating function. This not only reduces the wall clock time in practical applications but also promotes a lightweight technical implementation.

Analysis of the representation properties with synthetic and real-life application examples allows several interesting conclusions: (1) The proposed heatmap representation can approximate arbitrary curves/signals as long as no extreme curvatures (e.g., close to a directional change of $180^{\circ }$) are observed. (2) Calculating the heatmap with a mesokurtic function and small or medium overall width is preferred for most signal/curve types. Especially for filigree structures, it is advantageous to assume a low position uncertainty of the curve in space. (3) Even in the case of degraded image quality caused, for example, by shadowing, occlusion, or low SNR, the estimation of the heatmap by a CNN is robust and sufficiently accurate. Depending on the severity of the degradation, missing image information can be fully or partially extrapolated. (4) Both structures that are displayed directly in the image as well as structures derived from context can be approximated well. (5) The application to a 3D detection task shows encouraging results, although at a higher overall error when compared to heatmaps in 2D.

Comparing the heatmap representation to previous techniques allows us to derive advantages, drawbacks, and implications for practical applications. While similarly expressive and flexible as (binary) segmentation techniques, heatmaps inherently allow to incorporate prior knowledge of the expected signal distribution and its positional noise. In practice, this can be valuable for signals that expose some inherent structure of their orthogonal profile, e.g., a hollow-core metal rod observed via transmissive imaging. Moreover, we can more precisely represent structures with known physical parameters without having to perform explicit physical modeling, e.g., using the degree of stiffness/flexibility to determine plausible deviations of the nominal curve position. In contrast to related approaches, the proposed representation model also allows for instance-level parameterization of multiple individual structures within the same output channel. Furthermore, data augmentation can be applied to the numerical sparse point representation, eliminating interpolation noise observed in spatial transformations of the optimization target, e.g., a segmentation mask. An inherent downside of the approach is that the visual appearance of a signal with varying width (e.g., naturally non-uniform signals, perspective changes due to out-of-plane components, etc.) cannot be accurately modeled. While the learning algorithm can, to some degree, generalize for these cases, purely spatial methods like segmentation are much more suited for such tasks. Furthermore, for a dense accumulation of elongated structures with potential overlap, the method becomes much less efficient than a single-shot global representation like most segmentation methods.

In addition to these drawbacks, we want to note that no analysis has been performed to date on a suitable method that yields a parametric reconstruction of the original signal from the heatmap representation. Although a thinning approach yields satisfactory results in the pixel/voxel space, obtaining a functional description is not trivial, especially if the heatmap is fragmented or self-intersecting. This problem further intensifies if several heatmaps are close to each other and are approximated in the same output channel of a neural network. Consequently, separating structures into separate output channels would be desirable, even if no matching of object instances between multiple input images/volumes is possible. Also, using a neural network to directly predict the knots and parameter values of B-spline curve approximations^64^ is an attractive direction for future extensions of this work.

## Methods and materials

### Signal extraction and evaluation metric

Two post-processing protocols are used for signal recovery and empirical evaluation, depending on the curve type and the respective dataset: (1) Center-line extraction by medial axis transformation and subsequent comparison of the ASSD between ground truth and prediction curves. (2) Non-maximum suppression of the heatmap, morphological thinning, and subsequent calculation of the F-measure between ground truth and prediction curves.

#### Variant 1: ASSD

A signal estimate $C_{\text {pred}}$ is recovered as a set of points that satisfy the equality in (1) using the subsequent post-processing scheme: 1) Thresholding of the estimate with Otsu’s method^65^ to obtain a binary image. (2) Medial axis transformation to obtain a curve representation of unitary width. To this end, we use a thinning of the binary estimate with the skeletonization method by Zhang et al.^66^.

To estimate the quality of fit of such estimate, we use the ASSD metric^67^. A distance measure $d$ is defined as the minimum Euclidean distance of a point ${\varvec{x}}$ on a curve $C$ to all points ${\varvec{x}}^{\prime }$ on a second curve $C^{\prime }$, which is computed by $d({\varvec{x}}, C^{\prime }) = \min _{{\varvec{x}}^{\prime } \in C^{\prime }} \Vert {\varvec{x}}-{\varvec{x}}^{\prime } \Vert _{2}$. The ASSD between the two curves $C$ and $C^{\prime }$ is then calculated by evaluating this distance for every point ${\varvec{x}} \in C$ and ${\varvec{x}}^{\prime } \in C^{\prime }$ and averaging the resulting distance values

$$\begin{aligned} \text {ASSD} = \frac{1}{|C |+ |C^{\prime } |} \left( \sum _{{\varvec{x}} \in C} d({\varvec{x}}, C^{\prime }) + \sum _{{\varvec{x}}^{\prime } \in C^{\prime }} d({\varvec{x}}^{\prime }, C) \right) \end{aligned}$$

In theory, this evaluation policy has the advantage of being invariant to the width and distribution of the simulated signal and estimated heatmap. However, it has to be noted that the employed thinning can cause branching artifacts in case of noisy and self-intersecting heatmap estimates. Using a larger signal or heatmap width also implicates a higher upper limit of the possible length of such subsidiary branches. However, a qualitative review of the high-error cases revealed that this effect is negligible as most errors are caused by signal fragmentation in ambiguous regions.

#### Variant 2: F-measure

To conform to common evaluation protocols in boundary detection and skeletonization, we interpret the heatmap as a noisy curve probability map. First, a standard non-maximum suppression (NMS) is used to suppress curve point estimates for which a relatively stronger estimate is observed in orthogonal direction within some radius $r_{\text {spr}}$^68^. Second, the probabilities are converted to binary curve labels using some probability threshold. Third, iterative morphological thinning is employed until an edge of unitary width is obtained. The curve estimate is then compared to the ground truth curve by calculating the minimum-cost correspondence (i.e., Euclidean distance), evaluating it against a maximum distance tolerance $d_{\text {max}}$, and computing the F-measure $F=2\,PR/(P+R)$, where P and R mark the Precision and Recall, respectively^46^. Final reporting is done using the best curve selection threshold across all images in the dataset, i.e., at optimal dataset scale (ODS) or at optimal image scale (OIS)^69^. Individual configurations of $r_{\text {spr}}$ and $d_{\text {max}}$ are provided in Sect. “Datasets and training protocols”.

### Neural network architecture

For the 2D experiments, we use a single Hourglass Module which is a standard model for heatmap-based landmark detection^70^. The model is configured with a feature root of 128 used across all abstraction levels, ReLU activation functions in the bottleneck blocks, and instance normalization layers to obtain a smoothed optimization landscape in the presence of different contrast levels^71^. The bottleneck blocks are constructed in the standard 3-layer structure with half the number of convolution kernels in the actual bottleneck mapping. To facilitate true identity mapping without applied non-linearity, we use a pre-activation layout according to He et al.^72^. The 3D architecture is specified in Sect. “Surgical implants in CBCT volumes”.

The network parameters are initialized with the He initialization strategy^73^. The cost function operates on the output of the last layer and performs a heatmap-matching between network prediction and generated ground truth data. The default choice for the cost term is MSE, which is replaced by BCE in the segmentation experiments in Sect. “CNN-based estimation error for different signal and heatmap configurations”. Implementation was done in PyTorch v1.6.0^74^ (Python v3.8.5, CUDA v11.0, cuDNN v7.6.5). All experiments were performed on an x64 machine with an NVIDIA Titan RTX GPU and 64 GB memory. Details on data augmentation, data input standardization, and the remaining task-specific hyper-parameters are provided in the dataset descriptions in Sect. “Datasets and training protocols”.

### Datasets and training protocols

#### Simulated signals on chest X-ray

We analyze the proposed representation’s characteristics for different signal and heatmap configurations using simulated signals superimposed on chest X-ray images. First, a simple heuristic generation model is devised for the simulation of realistic curve signals. Therefore, a finite number of data points ${\textbf{D}}_{i}$ with $|{\textbf{D}}_{i} |\sim {\mathscr {U}}[3,10)$ is spatially distributed on a zero-valued envelope of size $\text {H:}256 \times \text {W:}256\,\textrm{px}$. The location of the seed point ${\textbf{D}}_{0}$ is randomly initialized such that it lies within a slightly more narrow sub-region to avoid too short and truncated curves: ${\textbf{D}}_{0;x},\,{\textbf{D}}_{0;y} {\mathop {\sim }\limits ^{i.i.d.}}{\mathscr {U}}[10,246)$. Based on this seed point, the $x, y$ coordinates of the subsequent points are calculated as ${\textbf{D}}_{i+1;x} = {\textbf{D}}_{i;x} + s\cos {\phi }$ and ${\textbf{D}}_{i+1;y} = {\textbf{D}}_{i;y} + s\sin {\phi }$ with $s \sim {\mathscr {U}}[20,60)$ and $\phi \sim {\mathscr {U}}[-90,90)$. If a newly sampled point exits the sub-region, a new seed point is generated, effectively sampling a new data point distribution. The parameters of the simulation model were chosen heuristically such that realistic global linear and angular moments are preserved over the point distribution. This promotes a minimum amount of stiffness and directional flow of the parameterized curve, which can be assumed for most real-life elongated structures such as surgical catheters or trivial skeletons. Upon the optimization of neural network parameters, the signal data points are simulated on the fly to maximize the number of curve characteristics and aid subsequent generalization. This online simulation is subject to a fixed global random seed to ensure comparability between different configurations. The data points that are used for validation are predefined and not changed between epochs or configurations.

Using the signal simulation model, we calculate a heatmap representation $H_{\text {sim}}({\varvec{x}})$ of the signal to be superimposed on a realistic background image $X_{\text {img}}$ (configuration provided in Supplementary Material Table S8). Upon fusion of signal and image, the heatmap is multiplied by a strength factor $a$, and an i.i.d. sampled additive Gaussian noise $Z$ is added. We obtain the final simulated image as $X_{\text {sim}} = X_{\text {img}} + \left( a \cdot H_{\text {sim}}({\varvec{x}})\left( 1 + Z\right) \right)$ with $Z \sim {\mathscr {N}}(0,\sigma _{Z}^{2})$. The background images stem from publicly available chest X-ray data^75^ originating from the *COVID-19 image data collection*^76^ and *ChestX-ray8*^77^ datasets. The 1125 images were partitioned into two cohorts for training and validation following a 900/225 ($80\%/20\%$) split. For comparison and reporting, we evaluated the metrics using the best model parameters for each signal/heatmap configuration directly on the validation dataset using the ASSD metric (Sect. “Variant 1: ASSD”).

Optimization is done with an Hourglass model for 40 epochs, a batch size of 2, a learning rate of 0.00025, and L2 regularization with a factor of 0.00005 using the RMSProp update policy. The number of interpolation points for polyline sampling is set to 200. An online augmentation sequence is applied to the training images, which involves minor shearing/axes shift i.i.d. sampled from $[-5,5]\,\textrm{px}$, rotation in the range of $[-5,5]^{\circ }$, image scaling by a factor of $[0.9,1.1]\,\%$, and a center crop which truncates $[0.0,0.1]\,\%$ of the height or width for each image side independently. Before training and inference, each image is brought to a spatial resolution of $\text {H:}256 \times \text {W:}256\,\textrm{px}$ by center-cropping the larger dimension w.r.t. the target aspect ratio followed by a cubic down-sampling.

#### Anatomical structures on knee radiographs

Network optimization and evaluation are performed on 223 clinical X-ray images retrospectively collected from anonymized databases. The images stem from different patients, are centered on the knee joint, and were acquired as strictly lateral projections with both distal femoral condyles superimposed with closely aligned contours. A medically trained engineer annotated the ground truth line segments corresponding to each anatomical structure with the labelme annotation tool^78^. A representative subset of 38 ground truth annotations was reviewed and verified by three expert trauma surgeons. The resulting dataset was split into two cohorts of 174/49 ($80\%/20\%$) for training and validation with disjoint patients. For the method comparison, we evaluate the metrics using the best model state on the validation dataset without separate analysis on a hold-out test dataset. We separate optimization between *direct* and *contextual* structures (Supplementary Material Fig. S12) using two separate Hourglass networks (Sect. “Neural network architecture”). The training was done for 250 epochs with a batch size of 2, a learning rate of 0.00025, and L2 regularization with a factor of 0.00005 using the RMSProp update policy. As each anatomical structure is always present in the image and has a unique appearance, we do not combine the structures into a single heatmap but rather use three distinct output channels for the direct structures. To aid generalization, the augmentation pipeline described in Sect. “Simulated signals on chest X-ray” is extended by horizontal flipping. A standardized training and evaluation resolution of $\text {H:}256 \times \text {W:}256\,\textrm{px}$ is obtained by resizing (retaining the aspect ratio) and a final center-crop. To evaluate potential effects of strong local curvature, e.g., for the Blumensaat line, we compare curve approximation variants with ($\text {S}>0$; *smooth*) and without ($\text {S}=0$; *close*) smoothing condition (Sect. “Numerical approximation and implementation”). Method evaluation is done with the ODS F-measure (Sect. “Variant 2: F-measure”). The distance tolerance for matches between edge predictions and ground truth is set to a standard value of $d_{\text {max}}=0.0075$ w.r.t. the image diagonal^69^. The suppression radius during NMS is set to $r_{\text {spr}}=1$.

#### Surgical implants in CBCT volumes

The dataset consists of 141 3D CBCT volumes recorded across a variety of different body regions: acetabulum, calcaneus, cervical/thoracal/lumbar spine, humerus, distal tibia, proximal tibia, and wrist. Each volume was acquired with a spatial resolution of $\text {L:}512 \times \text {H:}512 \times \text {W:}512$ voxels of size $0.31\,\textrm{mm}$ and was down-sampled to $\text {L:}128 \times \text {H:}128 \times \text {W:}128$ voxels to fulfill the constraints of available hardware. Each volume contains between 1 and 16 metallic screws or k-wires inserted into the bone for fragment fixation, ligament reconstruction, or plate osteosynthesis. The corresponding 3D ground truth volumes were generated according to the 3D extension explained in Sect. “Numerical approximation and implementation” and are based on two control points obtained by manual annotation of both the head and tip of each screw/wire. The data is split into training and validation cohorts of 101 and 40 volumes, respectively. Training and validation is done for six different reconstructions of each volume, differing in the size of the Gaussian reconstruction filter ($\sigma _{\text {rec}}\in [1,2,4]$) and in the application of a metal artifact reduction (MAR) technique (frequency-split MAR (FSMAR)^79^ and no MAR). Optimization is performed for 50 epochs using a 3D U-Net architecture^80^ with residual connections within each encoding and decoding block. The feature root is set to 16 and is doubled at each of the five encoder levels. We use a batch size of 1 and a learning rate of 0.005. Since each patient’s implant configuration differs, a single output heatmap volume is optimized. The ASSD metric (Sect. “Variant 1: ASSD”) is used to evaluate the quality of the reconstructed centerline of the screw/wire.

### Ethics approval

The proprietary clinical data was obtained retrospectively from anonymized databases and was not generated intentionally for the study. For this study type, formal consent is not required. All other data used within this study originates from publicly available datasets.

### Supplementary Information

Supplementary Information.

## Data Availability

The main data supporting the results in this study are available within the paper and its Supplementary Information. Primary and secondary datasets are disclosed as follows. (Primary datasets)—the simulated polylines used for analysis on synthetic data are available from the corresponding author, F.K., upon reasonable request. The 3D CBCT volumes and 2D anatomical structures data are not publicly available due to them containing information that could compromise intellectual property of Siemens Healthcare GmbH. (Secondary datasets) – all secondary datasets are publicly available. At the time of publication, the they can be downloaded from official repositories as follows: The Berkeley Segmentation Dataset 500 (BSDS500) and evaluation suite is available at https://www2.eecs.berkeley.edu/Research/Projects/CS/vision/bsds/. A pre-processed version of NYU Depth Dataset V2 (NYUD) following the data protocol in Gupta et al.^81^ and Xie & Tu^69^ is available at http://mftp.mmcheng.net/liuyun/rcf/data/NYUD.tar.gz. Skeletonization data for WH-SYMMAX is available at http://data.kaizhao.net/projects/skeleton/wh-symmax.zip, for SK-LARGE at http://data.kaizhao.net/projects/skeleton/sk1491.tar.gz, and for SK506 at http://data.kaizhao.net/projects/skeleton/sk506.tar.gz. The chest X-ray data can be obtained from https://github.com/muhammedtalo/COVID-19. The DeepGlobe Road Extraction Challenge dataset is available at https://competitions.codalab.org/competitions/18467. The TuSimple Lane Detection Challenge is available at https://github.com/TuSimple/tusimple-benchmark/issues/3.

## Code availability

The programming code used in this study is not publicly available due to it compromising intellectual property of Siemens Healthcare GmbH. The dataset-agnostic code for generation of 2D and 3D heatmaps of this study are available from the corresponding author upon reasonable request.
