# Supplementary material for: A principled representation of elongated structures using heatmaps
Source: Sci Rep. 2023 Sep 14;13:15253. doi: 10.1038/s41598-023-41221-2 (PMC10502041; doi:10.1038/s41598-023-41221-2)
Supplement: Supplementary file 1 — Supplementary Information. [file 41598_2023_41221_MOESM1_ESM.pdf]

Supplementary Information for

A Principled Representation of Elongated Structures using Heatmaps

Florian Kordon[1,2,3*], Michael Stiglmayer[4], Andreas Maier[1,2], Celia Martín Vicario[1], Tobias Pertlwieser[1], Holger Kunze[3,1]

[1]Pattern Recognition Lab, Friedrich-Alexander Universität Erlangen-Nürnberg, Martensstr. 3, Erlangen, 91058, Germany
[2]Erlangen Graduate School in Advanced Optical Technologies (SAOT), Friedrich-Alexander Universität Erlangen-Nürnberg, Paul-Gordan-Straße 6, Erlangen, 91052, Erlangen
[3]Advanced Therapies, Siemens Healthcare GmbH, Siemensstraße 1, 91301, Forchheim
[4]Optimization Group, Institute of Mathematical Modelling, Analysis and Computational Mathematics, University of Wuppertal, Gaußstr. 20, Wuppertal, 42119, Germany

*Correspondence should be addressed to Florian Kordon (florian.kordon@fau.de)

Contents

S1 Overview of dataset and method properties 3

S2 Susceptibility to different signal-to-noise ratios 4
S2.1 Experimental setup 4
S2.2 Results and discussion 5

S3 Object Contour and Edge Detection: BSDS500, NYUD 7
S3.1 Design and Setup 7
S3.1.1 Berkeley Segmentation Data Set 500 (BSDS500) 7
S3.1.2 NYU-Depth V2 (NYUD) 8
S3.2 Results and Discussion 8

S4 Skeletonization: WH-SYMMAX, SK-LARGE, SK506 10
S4.1 Design and Setup 10
S4.2 Results and Discussion 12

S5 Line Detection: TuSimple Lane Detection Challenge 15
S5.1 Design and Setup 15
S5.2 Results and Discussion 15

S6 Segmentation: DeepGlobe Road Extraction 18

S6.1 Design and Setup 18
S6.2 Results and Discussion 18

S7 Comparison to similar Gaussian representation and implementation variants **20**

S8 Experiment and training configurations **21**

S9 Complementary figure and tables **22**

S1 Overview of dataset and method properties

Table S1 Overview of exemplary datasets/tasks and different approaches for representing the contained elongated structures. For each approach, their principle applicability is marked.

**Dataset**	**Representation approach**					
	Binary seg.	Skelet.	Pose estim.	Line detect.	Polyline reg.	Heatmap reg.
Object contour						
BSDS500 [1]	✓	(✓) [1,2]	✗	✗	✗	✓
NYUD [2]	✓	(✓) [1,2]	✗	✗	✗	✓
Skeleton/Center-line						
WHSymMax [3]	✓	✓	✗	✗	✗	✓
SK506 [4]	✓	✓	✗	✗	✗	✓
SK-Large [5]	✓	✓	✗	✗	✗	✓
Straight lines						
Long bone axis	✓	✓[1]	✓	✓	✓	✓
3D CBCT implants	✓	✓[1]	✓	✓	✗	✓
Curved lines						
Anatomical features	✓	✓[1]	✗	✗	✓	✓
TuSimple [6]	✓	(✓) [1,2]	✗	✓	✓	✓
Mixed line styles						
DeepGlobe [7]	✓	(✓) [1,2]	✗	✗	✗	(✓)

[1] Boundary-agnostic methods.
[2] Amount and proximity/overlap of curve instances limits applicability.

Table S2 Overview of the considered datasets and the characteristics of the contained elongated structures. (✓: applicable, (✓): applicable under conditions, ✗: not applicable).

	Properties of elongated structures				
**Dataset**	Non-zero curvature	Significant step	Fixed shape	Occlusion	Variable object count
Straight lines					
Long bone axis	✗	✗	✓	✓	✗
3D CBCT implants	✗	✓	✓	✗	✓
Curved lines					
Anatomical features	✓	✓	✓	✓	✗
TuSimple [6]	✓	(✓) [3]	✗	✓	✓
Object contour					
BSDS500 [1]	✓	(✓) [1]	✗	✗	✓
NYUD [2]	✓	(✓) [1]	✗	✗	✓
Skeleton/Center-line					
WH-SYMMAX [3]	✓	✗	✗	✓	(✓) [2]
SK506 [4]	✓	✗	✗	✓	(✓) [2]
SK-LARGE [5]	✓	✗	✗	✓	(✓) [2]
Mixed line styles					
DeepGlobe [7]	✓	✓	✗	✗	✓

[1] Depends on edge type (depth, reflectance, etc.) and image modality (HHA/RGB) [8].
[2] Multiple skeleton branches for non-trivial objects.
[3] Affected by road wear and shadowing.

S2 Susceptibility to different signal-to-noise ratios

The visibility/strength of a signal is a critical factor in whether a CNN can infer a meaningful heatmap representation. To elaborate on this behavior in greater detail, we simulate various signal shapes on a noise background subject to a fixed signal-to-noise ratio (SNR). Based on previous analysis, we assume each analyzed distance-dependent function to be approximately equally affected by the level of noise. For that reason, we only examine signals and heatmaps of Gaussian distribution as a representative selection.

S2.1 Experimental setup

The signals are subjected to a Gaussian background noise governed by different SNR ratios. The SNR represents the ratio of the mean power of the effective signal to the mean noise power of the interfering background signal and can be formally defined as $\mathrm{SNR} = A_{\mathrm{signal}}/\sigma_{\mathrm{noise}}$. Using this relation, we can calculate the required overlay strength a for various SNR ratios if we keep the parameterization of the noise signal fixed.

$$X_{\mathrm{sim}} = Z_{\mathrm{img}} + \left(A_{\mathrm{signal}} \cdot H_{\mathrm{sim}}(\boldsymbol{x})\left(1 + Z_{\mathrm{signal}}\right)\right)$$

Z_{img} marks the background noise signal with $\sigma_Z = 0.1$ and $Z_{\text{img}} \sim \mathcal{N}(0, \sigma_Z^2)$. Z_{signal} represents some direct signal deterioration with $\sigma_Z = 0.1$.

The signals are constructed using the same simulation model from the experiment "Simulated signals on chest X-ray" in the main article. Individual models are trained for SNR $\in \{0.25, 0.50, \ldots, 2.50\}$, different widths of the simulated signals given by $\sigma_{\text{sim}} \in \{1, 3, 5\}$, and different heatmap widths given by $\sigma_{\text{hm}} \in \{1, 3, 5\}$. All models are compared using the ASSD metric.

S2.2 Results and discussion

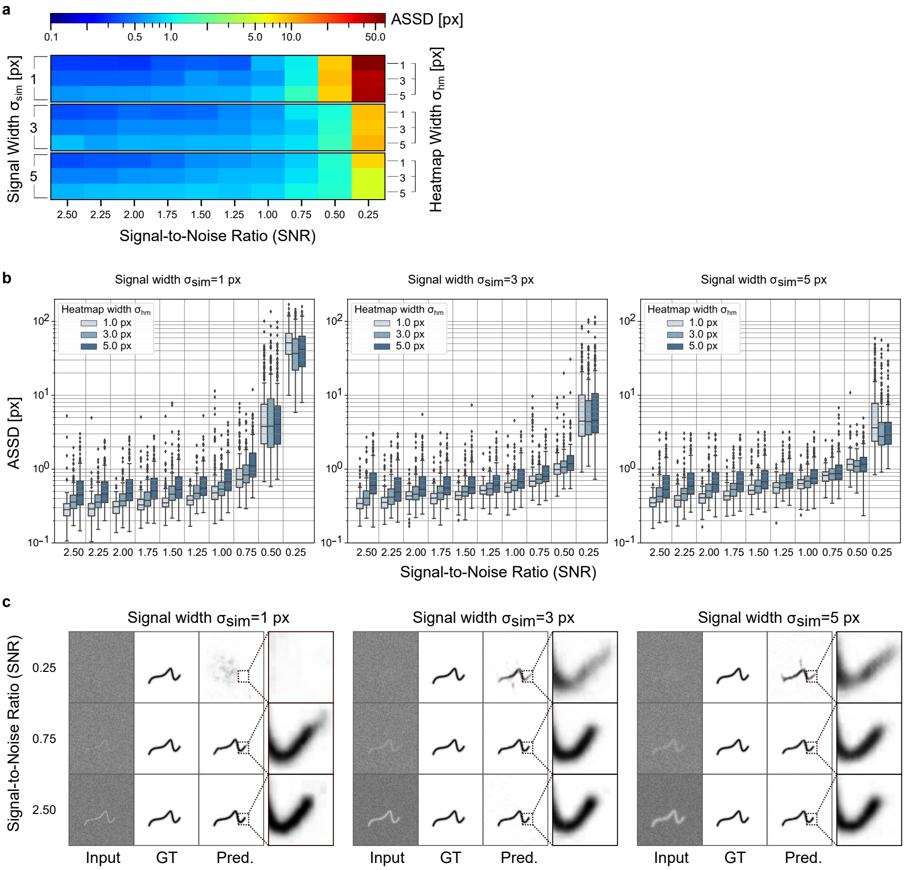

Fig. S1 Effect of the SNR level. **a**, heatmap visualization for different experiment configurations. **b**, Boxplot diagrams illustrating the error development for different SNR and signal/heatmap configurations. **c**, Illustration of heatmap prediction quality upon different SNR values at a heatmap width of $\sigma_{\text{hm}} = 3\,\text{px}$. The shown sample was selected according to the smallest accumulated difference to the mean values of the ASSD metric over all possible signal and heatmap configurations.

As shown in Fig. S1, an SNR of 0.75 suffices an adequate heatmap with a low representation error of the original signal. For an SNR below that threshold, the heatmap quality varies substantially with the composition of signal width and heatmap width. For such low SNR environments, wider signals (here $\sigma_{\text{hm}} \geq 3\,\text{px}$) can still recover most of the original signal, although a clear underestimation of the intensity values can be observed. More narrow signals ($\sigma_{\text{hm}} = 1\,\text{px}$) lead to a strong deterioration of the predicted heatmap, where often only a locally restricted noise in the signal proximity is estimated (Fig. S1). A relatively larger signal portion can easily explain this in the case of wide signals. Both the simulated signal noise and the noise/background signal have a proportionally less impairing influence on the reconstruction of the original signal trace. Similar to the observations in the main section, 'CNN-based estimation error for different signal and heatmap configurations,' the estimation with narrow heatmaps yields the lowest error of all evaluated signal widths. Only at very low SNR values the performance for different heatmap widths equalizes (for SNR = 0.50), or a slightly higher heatmap width is preferable (for SNR = 0.25).

Interestingly, the obtained error scores show only small improvements to the reconstruction quality if the SNR is increased beyond a value of 0.75. This indicates that the proposed heatmap representation of elongated structures is a suitable choice in noisy environments with medium to strong limitations to the visibility of the original signal. Although care should be taken when transferring this characteristic to real-life applications with significantly more complex signal and noise/background patterns, we assume this to be a valuable observation and characteristic of the proposed method.

S3 Object Contour and Edge Detection: BSDS500, NYUD

Edges or contours of salient objects are of fundamental interest for many vision and scene understanding tasks. Depending on the relevance and perceived saliency of different objects specified by the annotator, the contour map to be detected ranges from very sparse scenes to very dense and comprehensive edge relations. We explore the applicability of the proposed method to generic outdoor scenes on the widely used BSDS500 dataset [1] and dense indoor environments on the NYUD dataset [2]. We compare it with state-of-the-art detectors specifically tailored to this type of detection task and generic edge detection methods. The evaluation results are shown in Fig. S2, Tab. S3, Tab. S4, and Fig. S14, Fig. S15.

S3.1 Design and Setup

S3.1.1 Berkeley Segmentation Data Set 500 (BSDS500)

The *Berkeley Segmentation Data Set 500* (BSDS500) is a popular dataset for edge detection. It contains 500 natural RGB images from various outdoor scenes split into training (200), validation (100), and test (200) cohorts. The ground truth edge annotations stem from 4-9 annotators, differing in the level of contour sparsity and the number of perceived relevant objects per image. Since the boundaries of non-trivial objects cannot be conceived as a single elongated structure, we define a parametric curve description for each boundary branch. For that purpose, each branch's path coordinates are determined by interpreting the boundary lines as a skeleton graph and analyzing branching points. The polyline for each skeleton branch is used to generate a partial heatmap. These partial heatmaps are finally converted into a single boundary map using a maximum-intensity projection.
The boundary heatmaps for every annotator ground truth are averaged to generate a single probability map. In contrast to other recent works [9, 10], we do not discard edge positions that are below a certain probability threshold. For training, we use a deeply supervised U-Net++ [11] with deep supervision at multiple feature scales for additional semantic constraints between low- and high-level features. The final optimization cost is calculated as the linear sum of all individual cost terms. Here, we use the original network structure with a feature root of 64, batch normalization layers, and trainable skip connections.. To enable processing at the original image resolution and account for various aspect ratios, we train the network using randomly sampled patches of size H:256 $\times$ W:256 px, four patches per image (sampled once per epoch), and a total of 200 patches per epoch. For patch aggregation, the patches are sampled with 128 px overlap along each dimension and fused using value averaging. Optimization is done for 50 epochs, a batch size of 2, a learning rate of 0.00025, RMSProp policy, and a learning rate decay after 40 epochs by a multiplicative factor of 0.1. The number of interpolation points for polyline

sampling is set to 50. We use the same data augmentation as [12] for the training cohort training in an online manner, including rotation and flipping. For evaluation we report the ODS and OIS F-measure with standard values $d_{\max} = 0.0075$ w.r.t. the image diagonal and $r_{\text{spr}} = 2$ [12].

S3.1.2 NYU-Depth V2 (NYUD)

The *NYU-Depth V2* (NYUD) combines 1449 three-channel RGB images and corresponding depth information with pixel-wise annotations of semantic classes [2]. Similar to BSDS500, it is commonly used for dense edge detection following the edge extraction, and data preprocessing scheme in Gupta et al. [13]. Accordingly, the data is split into training (381), validation (414), and test (654) cohorts [13]. To incorporate the depth information, HHA features are used, which separate the depth values into a three-channel image representation of horizontal disparity (H), height above ground (H), and angle of the local surface normal with the inferred direction of gravity (A) [12]. We use the same optimization and data processing scheme as described in Sect. S3.1.1 and employ the augmentation scheme described in [12] on the extracted patches. Training is performed separately on the RGB and HHA images, and the two boundary heatmap predictions are averaged for algorithm evaluation [9, 12]. The correspondence tolerance is increased to $d_{\max} = 0.011$ w.r.t. the image diagonal and the NMS radius is set to $r_{\text{spr}} = 4$ to account for larger image sizes [12, 14].

S3.2 Results and Discussion

BSDS500. We compare the performance of our approach on the BSDS500 dataset with several classical detection algorithms like Canny [15], Pb [16], SE [17] and OEF [18], as well as recent deep learning detectors like HED [12], RCF [9], DeepContour [19], DeepEdge [20], HFL [21], DDS [22], and others. Representing object boundaries as heatmaps leads to an ODS F-measure of 0.749, which is comparable to early Deep Learning based edge detectors, but falls short compared to specialized edge detectors like HED [12] and recent highly optimized methods like RCF [9] and AFPN-NMS [10]. The PR curves in Fig. S2-**a** reveal a systematically lower recall than comparable methods, indicating that not all object edges are detected reliably. These issues can be alleviated using multi-scale inference, additional training data (e.g., the Pascal Context dataset [23]), and a network architecture explicitly tailored for this task type.

NYUD. The performance on the NYUD dataset is compared to that of several methods, including OEF [18] (only operating on RGB images), HED [12], RCF [9], and others. Averaging the detections on the RGB and HHA image cohorts yields an F-measure of 0.739, which is comparable to the performance of the HED detector [12]. The substantial performance improvement upon fusion of RGB and HHA edge predictions suggests that both information types complement each other. In particular, using only the HHA features

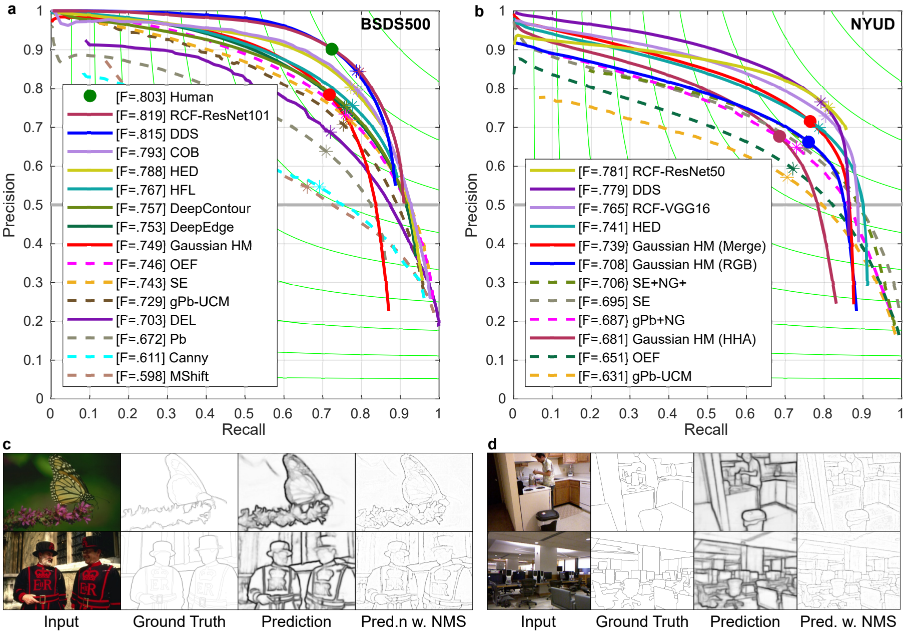

Fig. S2 Evaluation for boundary detection tasks. **a,c**, PR curves and predictions on BSDS500. **b,d**, PR curves and predictions on NYUD.

reveals problems for edges that materialize by discontinuities of illuminance or surface reflectance [8]. For very dense scenes with little gaps between object edges, the heatmap representation suffers an overlap of noise distributions along the curve responses, resulting in edge bleeding [17]. This overlap makes it difficult to delineate individual lines from the predicted edge maps and often results in diffuse line predictions that resemble the mean of several closely spaced edges.

Table S3 Comparison of the F-measure on BSDS500. Results for related methods are extracted from Liu et al. and Sun et al. [9, 10]. (†: Trained with multi-scale fusion and additional Pascal Context training dataset [23]).

	**BSDS500**	
Method	F-measure ODS	F-measure OIS
Canny [15]	0.611	0.676
Pb [16]	0.672	0.695
SE [17]	0.743	0.764
OEF [18]	0.746	0.770
gPb-UCM [1]	0.729	0.755
AFPN-NMS [10]	0.815	0.833
AFPN-NMS† [10]	**0.839**	**0.856**
RCF [9]	0.798	0.815
RCF† [9]	0.819	0.836
DDS† [22]	0.815	0.834
DEL [24]	0.703	0.735
COB [25]	0.793	0.819
HED [12]	0.788	0.808
HFL [21]	0.767	0.788
DeepEdge [20]	0.753	0.772
DeepContour [19]	0.757	0.776
Gaussian Heatmap		
$\sigma_{\text{HM}} = 1$	0.746	0.768
$\sigma_{\text{HM}} = 2$	0.749	0.770

S4 Skeletonization: WH-SYMMAX, SK-LARGE, SK506

We investigate whether the heatmap representation approach can also be used for skeletonization. Similar to the boundary detection tasks, a skeleton for non-trivial objects contains multiple branches and cannot be represented by a single line. For that reason, the skeleton is analyzed for branching points, and a parametric curve description is calculated for the component branch. A unified representation of the skeleton is obtained by merging the heatmaps of the individual branches using a maximum-intensity projection.

S4.1 Design and Setup

We evaluate the skeletonization performance on three benchmark datasets: 1) WH-SYMMAX [3], 2) SK-LARGE [5], SK506 [4].

To accommodate different aspect ratios and vastly different image resolutions, we follow a patch-wise training strategy and employ the U-Net++ network with additive deep supervision [11]. The optimization is performed over 120 epochs with a patch size of H:112 × W:112 px using 8 randomly sampled patches per image during each epoch. We use a batch size of 8 and a total of 800 batches per optimization epoch. As some SK-LARGE and SK506 images are of smaller spatial resolution than the patch size, we perform bi-linear

Table S4 Comparison of the F-measure on NYUD. Results for related methods are extracted from Liu et al. and Sun et al. [9, 10].

	NYUD	
Method	F-measure ODS	F-measure OIS
SE [17]	0.695	0.708
SE+NG+ [14]	0.706	0.734
OEF [18]	0.651	0.667
gPb-UCM [1]	0.631	0.661
gPb+NG [13]	0.687	0.716
AFPN-NMS [10]		
RGB	0.756	0.772
HHA	0.720	0.729
RGB+HHA	0.780	0.794
RGB+HHA (ResNet50)	**0.790**	**0.802**
RCF [9]		
RGB	0.743	0.757
HHA	0.703	0.718
RGB+HHA	0.765	0.780
RGB+HHA (ResNet50)	0.781	0.793
HED [12]		
RGB	0.717	0.732
HHA	0.681	0.695
RGB+HHA	0.741	0.757
Gaussian Heatmap ($\sigma_{\mathrm{HM}} = 2$)		
RGB	0.708	0.723
HHA	0.681	0.696
RGB+HHA	0.739	0.755

up-sampling during training (nearest neighbor for the ground truth skeleton map), maintaining the original aspect ratio. For aggregating the patches during evaluation/testing, we uniformly sample the patches with an overlap of H:64 $\times$ W:64 px in both dimensions and average the intensities in the overlap regions. Potentially up-sampled images and their predictions were resized to their original resolution during evaluation. The parameters were updated using an AdamW optimizer at a default learning rate of 0.001 and a learning rate decay after 80 epochs with a multiplicative factor of 0.1. We performed model selection using a random 10% split of the training data as a validation cohort. We use an online augmentation strategy on the training patches using horizontal flips $p = 0.5$ as well as rotation with $\alpha \in \{0, 90, 180, 270\}^\circ, p = 1.0$. The heatmap representation was constructed using a Gaussian function with $\sigma = 2$ px without normalization constant, 50 interpolation points per branch, and no spline smoothing to account for the densely labeled ground truth.

We follow the conventional evaluation protocol on the test set using a standard non-maximum suppression for skeletonization [17] and comparison of the predicted skeleton probability maps against the ground truth using the ODS F-Measure $F = 2\,PR/(P + R)$. Following standard practice, [26, 27], the maximum allowed distance tolerance for matches between the skeleton

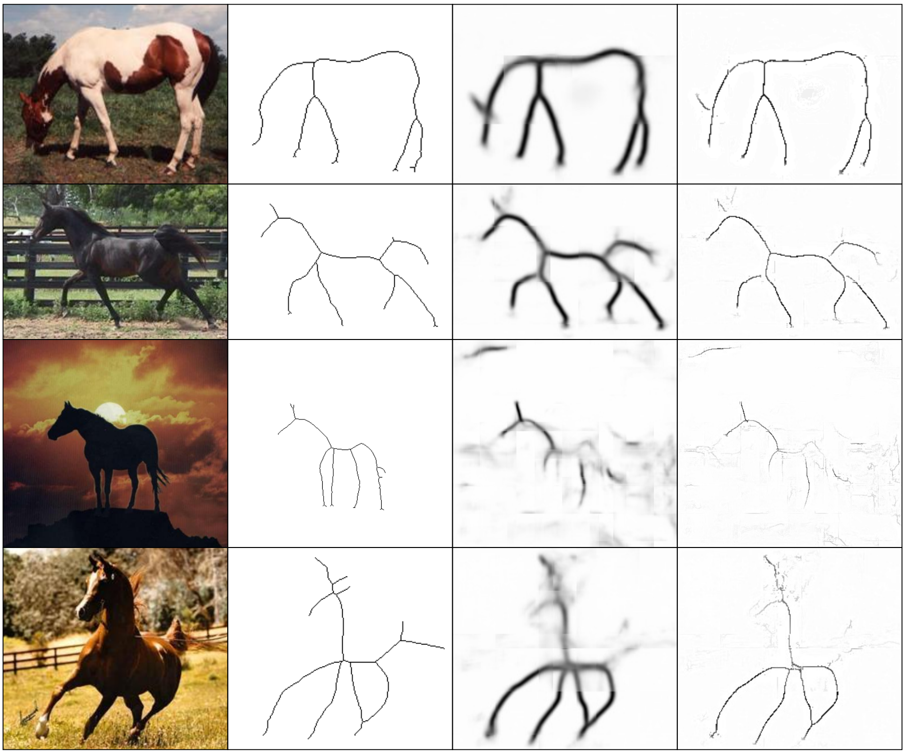

Fig. S3 Qualitative skeletonization analysis on WH-SYMMAX. Strong performance is observed for horses in good illumination and typical poses covered in the training cohort. The predicted heatmaps showed weak intensities in ambiguous regions for unseen viewing directions and foreground/background combinations, resulting in missing branches and frayed edges in the reduced skeleton.

prediction and ground truth is set to $d_{\mathrm{max}} = 0.01$ w.r.t. the image diagonal and the NMS radius is set to $r_{\mathrm{spr}} = 1$.

S4.2 Results and Discussion

The numerical results are listed in Tab. S5. The heatmap representation shows competitive performance compared to specialized skeletonization methods, and the HED [12] and RCF [9] edge detectors on WH-SYMMAX with mostly standardized object pose and viewing direction (Fig. S3), but is outperformed on the much more diverse and unconstrained SK-LARGE and SK506 datasets (Fig. S4). Across all datasets, the DeepFlux algorithm [26] performs best and surpasses boundary-aware and scale-regression approaches as well as the considered edge detectors by a big margin. The qualitative results reveal overall good performance on humans, animals, and objects in standard poses and good illumination but show less intensity and uncertain regions for objects in rare

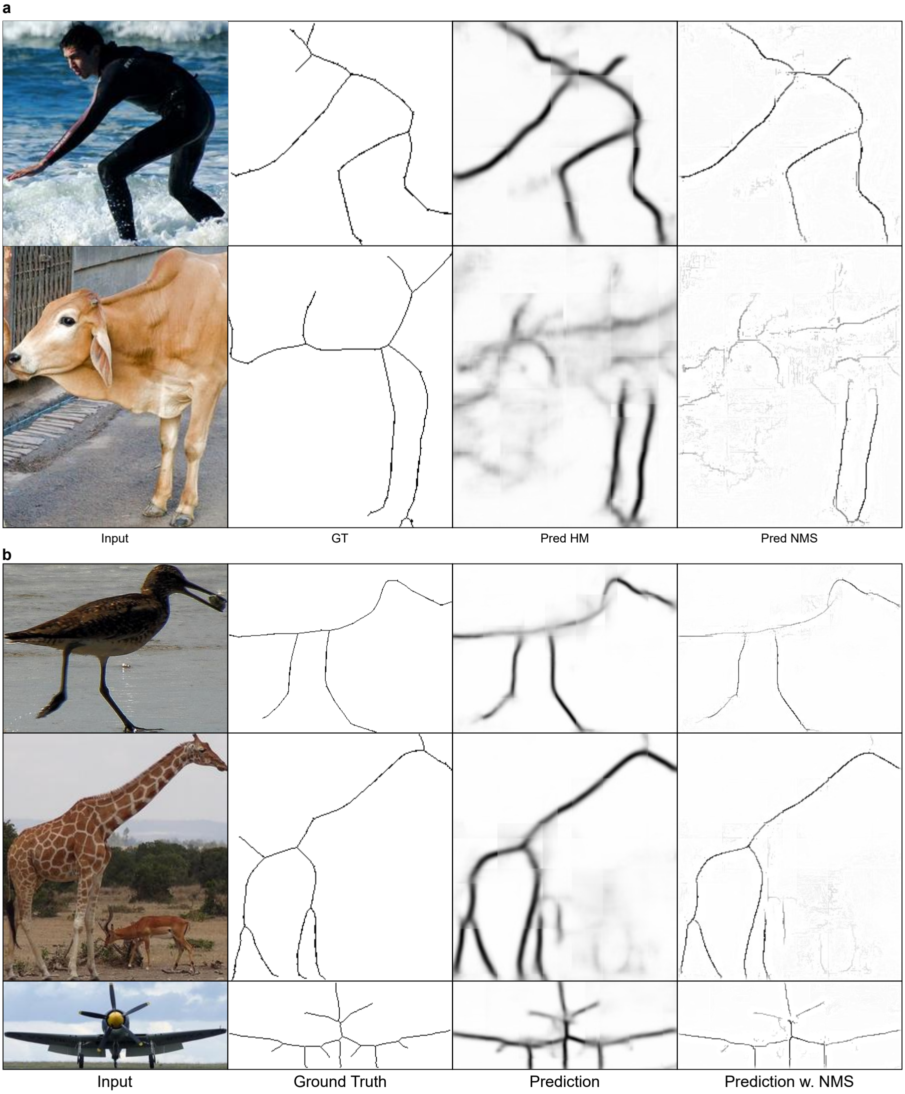

Fig. S4 Qualitative analysis for skeletonization in unconstrained outdoor scenes. **a**, examples for SK506, revealing problems for partially imaged objects and large regions which exceed the patch size. **b**, examples for SK-LARGE. The skeleton for smaller images with higher object standardization is consistently better than for objects with large pose variations.

viewing angels or objects with uncommon shapes and little to no surrounding context space.regression approaches by a big margin.

We argue that these issues, as well as the performance difference in general, can be largely explained by the lack of an explicit combination of high-level semantics with local high-resolution details. As pointed out by [27], the "semantic vs. resolution conflict" is commonly tackled by fusion of multiple

Table S5 Comparison of skeletonization performance using the ODS F-measure. The results for comparative approaches are extracted from Xu et al. [26].

**Method**	**WH-SYMMAX**	**SK-LARGE**	**SK506**
MIL [28]	0.365	0.353	0.392
HED [12]	0.732	0.497	0.541
RCF [29]	0.751	0.626	0.613
LMSDS [5]	0.779	0.649	0.621
FSDS [4]	0.769	0.633	0.623
SRN [30]	0.780	0.678	0.632
LSN [31]	0.797	0.668	0.633
Hi-Fi [27]	0.805	0.724	0.681
DeepFlux-P [26]	0.861	0.750	0.717
DeepFlux-E [26]	**0.867**	**0.754**	**0.719**
Gaussian Heatmap			
$\sigma_{\mathrm{HM}} = 1$	0.805	0.552	0.561
$\sigma_{\mathrm{HM}} = 2$	0.810	0.559	0.551

feature scales, which we have not considered in our training and evaluation protocol. In this context, it is worth noting that a patch-based approach is expected to perform worse in the case of large semantically cohesive regions. For such images, a larger patch size, a standardized resolution of the images where patches are sampled from, or an approach without patch-wise processing might be beneficial. DeepFlux [26]) tries to address the semantics conflict by incorporating a broader context by combining multiple vector estimates within a predefined context area. Having multiple estimates from different regions helps to stabilize the computed flux and the subsequent skeleton point prediction.

S5 Line Detection: TuSimple Lane Detection Challenge

We apply our method to lane detection on multi-frame recordings of road scenes using the *TuSimple Lane Detection Challenge* dataset [6]. The individual lanes should be estimated as a continuous curve, although they can be (partially) occluded by other vehicles in front of the recording vehicle.

S5.1 Design and Setup

The TuSimple dataset provides 7,000 1 s-short video clips of 20 frames shot from a front-facing camera system mounted on a moving car [6]. A polyline annotation for a maximum of 5 lanes with up to 48 control points is provided for the last frame of every clip in the training set (3626 clips). In contrast to the previously synthesized signals, we can not assume a fixed signal shape for the lane markings. Besides, the lane markings extend far away from the optical center resulting in a large depth range that is captured. Consequently, the signal width becomes significantly smaller as the respective vanishing point is approached. To address this challenge and support a mesokurtic function (e.g., Gaussian) as an adequate choice for configuring the heatmap ground truth for a new task, an CNN is trained individually for different configurations of the distance-dependent function and different heatmap widths. Based on learnings from the representation property analysis, the heatmap width is set to a small value with $\sigma_{\mathrm{hm}} \in \{1, 2, 3\}$ px at an image size of H:144 $\times$ W:256 px. A single ground truth map is generated as the maximum intensity projection of all individual lane heatmaps to support a variable amount of lane markings within each image. The corresponding augmentation policy resembles that of the dataset "Anatomical structures on knee radiographs" but replaces the affine rotation from the set of possible online augmentation steps with a horizontal flipping. Training is done for 100 epochs using an Hourglass model with a feature root of 64, a batch size of 2, a learning rate of 0.00025, and L2 regularization with a factor of 0.00005 using the RMSprop update policy. The number of interpolation points m is set to 50 to adjust for minor curvature in the lane markings. The annotated data is split into a training and validation fold with 2900/726 (80%/20%) images, respectively.

S5.2 Results and Discussion

Low ASSD errors for the heatmap predictions indicate a high quality of curve reconstruction, which suggests that the proposed representation is a good fit for the target task (Fig. S5, Fig. S6, Tab. S6. The DeepFlux skeletonization method suffers from an ambiguous vector field in regions where the context areas of individual road lanes overlap. Although a context of lower width can alleviate this problem, it also increases susceptibility to occlusion and shadowing, which ultimately decreases the detection accuracy. The distance-weighted

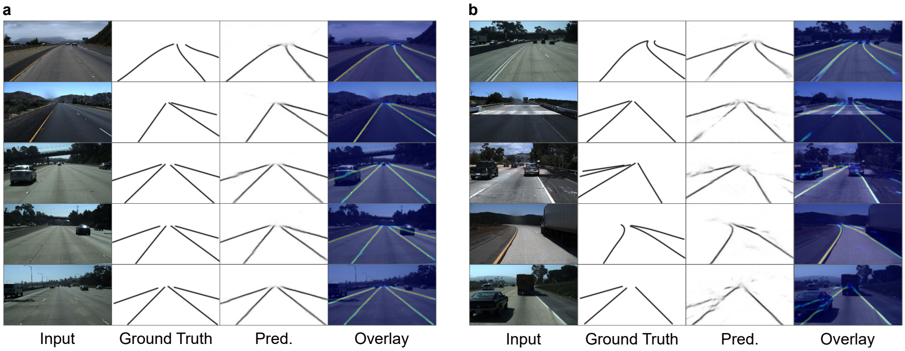

Fig. S5 Qualitative analysis on TuSimple dataset. **a**, shows low-error predictions. **b**, shows erroneous samples. The images were selected amongst the respective top/bottom 20 ASSD test fold scores with Gaussian distribution and $\sigma_{\text{hm}} = 1\,\text{px}$.

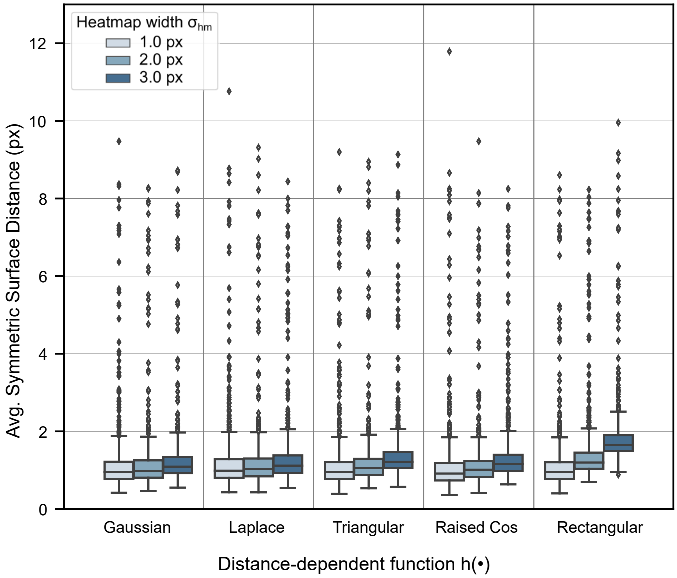

Fig. S6 Boxplot error distributions of different heatmap configurations for the task of lane marking detection. The most frequent reason for outliers is a false-positive heatmap response at side strips that are not part of the dataset annotations.

binary segmentation yields comparable results to the best-performing heatmap model.

The observations largely match the analyzed representation properties: a narrow and mesokurtic heatmap configuration yields the most consistent results. Also, there are no significant differences in the performance for the different distance-dependent functions except the rectangular choice, which shows a substantial error increase for larger heatmap widths. This strengthens the assumption that the heatmap distribution plays a subsidiary role. Similarly, a small heatmap width leads to homogenization of the different distributions.

Table S6 Lane detection performance of different representation methods on the TUSimple dataset measured with the ASSD metric [px]. While the DeepFlux methodology suffers from an ambiguous vector field in areas of meeting road lane markings, the distance-weighted segmentation and the heatmap approach yield similar performances.

**Method**	**Thr.**	**TuSimple**
BCE	0.5	25.64 ± 17.03
	Otsu	1.24 ± 0.99
Dice	0.5	2.26 ± 1.51
	Otsu	2.26 ± 1.51
BCE+Dice [32]	0.5	2.29 ± 1.51
	Otsu	2.29 ± 1.51
W4BCE [33]	0.5	1.24 ± 0.96
	Otsu	1.20 ± 0.98
DeepFlux-P [26]		
context=8	0.4	1.96 ± 1.35
context=10	0.4	1.50 ± 1.17
context=12	0.4	1.51 ± 1.21
context=14	0.4	1.41 ± 1.04
context=16	0.4	1.55 ± 1.14
Gauss. heatmap		
$\sigma_{\text{HM}} = 1$	Otsu	$\mathbf{1.18 \pm 0.99}$
$\sigma_{\text{HM}} = 2$	Otsu	1.21 ± 0.99
$\sigma_{\text{HM}} = 3$	Otsu	1.32 ± 0.97

For example, for a value of $\sigma_{\text{hm}} = 1\,\text{px}$, the width of the compact hull amounts to only 7 pixels, which implies a strong value-binning of the curve-orthogonal intensity profile.

In most cases, occluded lane markings are still inferred correctly using global contexts like elevation, standard lane orientation, and an almost static linear perspective. By that, partial visibility of a marking usually is enough to connect smaller visible segments and extrapolate at occluded areas. Nevertheless, several factors lead to erroneous predictions:

1. Spurious detection of side strip lane markings.
2. Strong occlusion.
3. Low contrast due to road damage, wear of the markings, or overarching structures like bridges or overpasses.
4. High variability of the marking appearance.

S6 Segmentation: DeepGlobe Road Extraction

We further analyze the general applicability of our approach to image segmentation. A fitting task is the detection of roads and street networks on satellite images. Major challenges are the great variability in shape, structure, width, texture, and contrast to the background.

S6.1 Design and Setup

Analogous to the analysis and preparation of the centerline during skeletonization (Supplementary Information S4), each road and path can be converted to a single, partial polyline. A single heatmap representation for the complete image can be generated by combining each heatmap using a pixel-wise sum. To create the final binary segmentation mask, Otsu thresholding is applied. We evaluate this process on the DeepGlobe Road Extraction challenge [7]. For that purpose, the images are resized to a standard resolution of H:256 $\times$ W:256 px. The heatmap width is set to $\sigma_{\mathrm{hm}} \in \{1, 2\}$ px. Online data augmentation involves horizontal and vertical flipping. For training, we used an Hourglass model with feature root 128. It was optimized for 120 epochs with a batch size of 8, a learning rate of 0.00025, and L2 regularization with a factor of 0.00005. The data was divided into training and validation cohorts with a 90%/10% split (5604/622 images).

S6.2 Results and Discussion

For the official challenge validation split consisting of 1243 images, the approach yields a mIoU score of 0.448. We observe a large performance gap to the evaluation on the internal validation split (622 images) for which a mIoU of 0.734 is achieved. Qualitative evaluation (Fig. S7) shows that single roads are detected with high precision. In contrast, a high amount of false-positive activation at small farm roads and side roads is observed for dense street networks. In addition, the ground truth generation pipeline does not account for the broad spectrum of road widths, which ultimately limits accuracy for very broad and very narrow instances. The problems can be alleviated by utilizing different heatmap widths for the individual street branches. Separating different road widths into separate output channels could also provide additional information in the subsequent semantic analysis in downstream tasks. Also, to lower the activation for small side roads that are not covered in the ground truth data, a patch-based approach that works on full-resolution images could aid in better differentiating different road types. However, a conventional segmentation approach is preferable for such dense prediction tasks where pixel-wise ground truth is available.

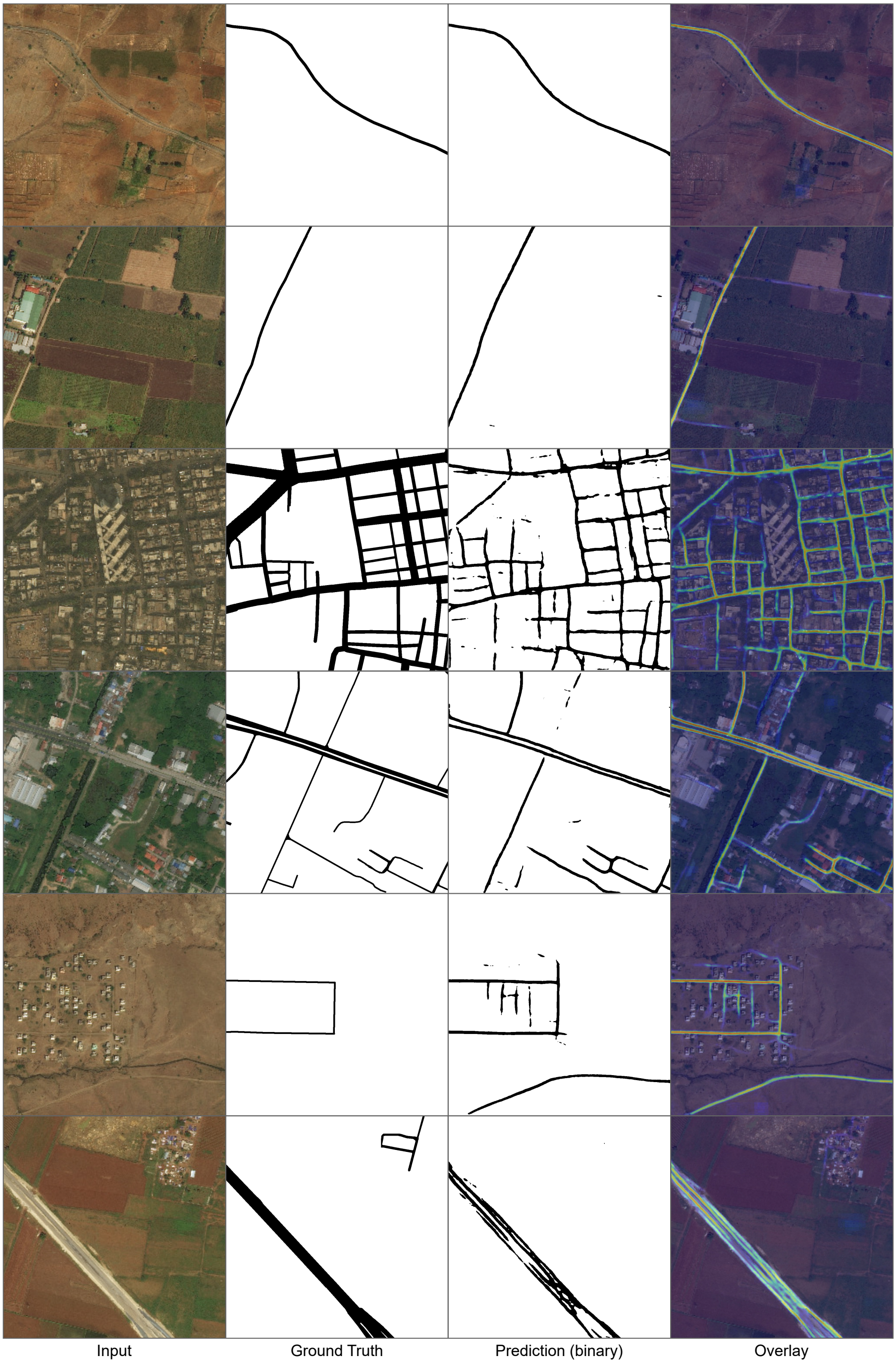

Fig. S7 Internal validation set samples for DeepGlobe Road Extraction data.

S7 Comparison to similar Gaussian representation and implementation variants

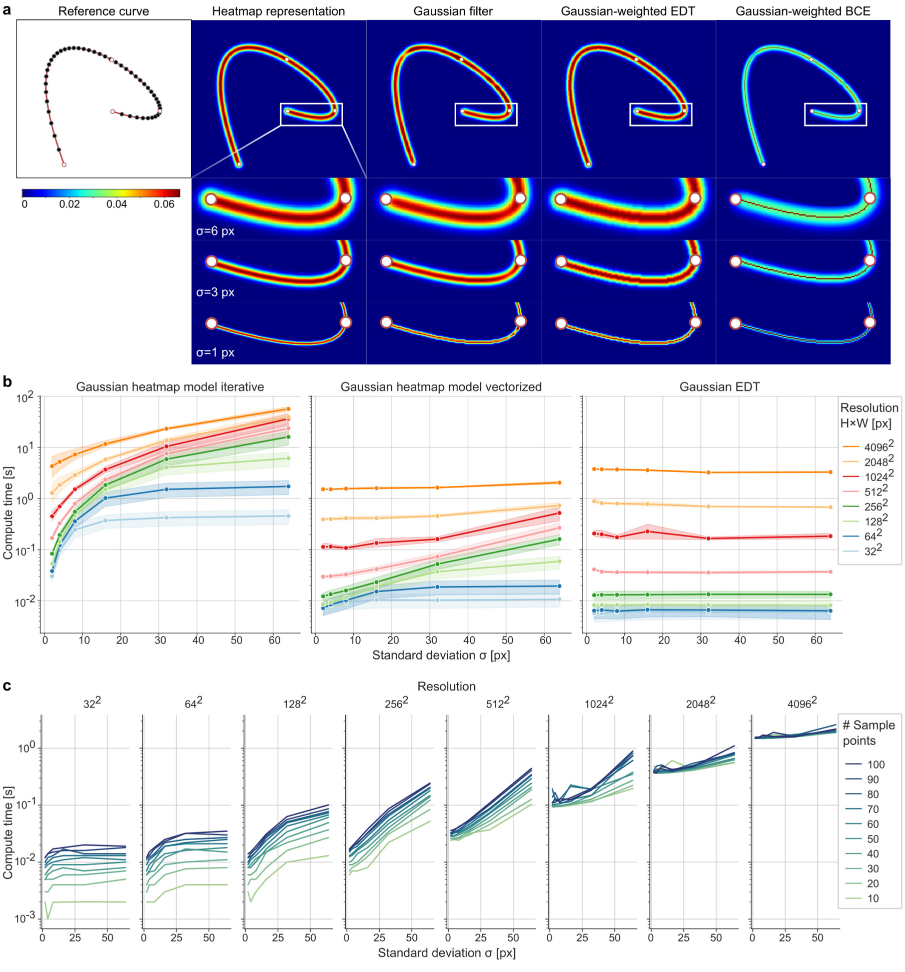

Fig. S8 Analysis of spatial accuracy and compute time of the heatmap representation and similar spatial curve representations. **a**, comparison of spatial accuracy for different heatmap widths on a medium-sized envelope H:401 × W:443 px. The Gaussian-weighted EDT variant shows step artifacts, and the Gaussian filter shows fluctuations of the curve intensity and noise profile due to different numbers of convolution operations per spatial location. The proposed heatmap representation and computation exploit sub-pixel information and yield a smooth curve approximation. **b,c**, comparison of compute time between the proposed method in iterative and vectorized form and Gaussian-weighted EDT.

S8 Experiment and training configurations

Table S7 Curve parameterization for estimation of heatmap error bounds. The number of possible curve expressions is given by the Cartesian product of the parameter set.

**Parameter**	**Sample space**
Distance-dependent function $h(\cdot)$	Gaussian
Reduction function $h^*(\cdot)$	Min. Distance, IDW ($p = \{1, 2\}$)
Envelope size	H:2000 $\times$ W:2000 px
Number of evaluation points $\lvert E_0 \rvert$	1000
Mean interpolation point distance	$\{2, 4, 8, \ldots, 256\}$ px
Knot configuration δ	{0, 0.1, ..., 1}
Heatmap width	
$\rightarrow$ as absolute margin width	$\{50, 100, \ldots, 400\}$ px
$\rightarrow$ as std σ of $h(\cdot)$	$\{8.5, 16, \ldots, 66\}$ px

Table S8 Configurations for both the simulated signals as well as heatmap representations. The number of possible curve expressions is given by the Cartesian product of the parameter set.

**Parameter**	**Sample space**
Envelope size	H:256 $\times$ W:256 px
# interpolation points m	200
Cost function	Mean Squared Error (MSE), Binary Cross Entropy (BCE) only for rect. heatmap
Simulated signal $H_{\text{sim}}(\boldsymbol{x})$	
Distance-dependent function $h(\cdot)$	Gauss., Laplace, Triang., Rect., Raised Cos., Cath. ($s = 0.75$)
Strength of linear overlay a	$\{0.15, 0.30\}$
Add. Gaussian noise $Z \sim \mathcal{N}(0, \sigma_Z^2)$	$\sigma_Z = 0.1$
Signal width	
$\rightarrow$ as absolute margin width	$\{7, 19, 31\}$ px
$\rightarrow$ as std σ_{sim} of $h(\cdot)$	$\{1, 3, 5\}$ px
Heatmap $H_{\text{hm}}(\boldsymbol{x})$	
Distance-dependent function $h(\cdot)$	Gauss., Laplace, Triang., Rect., Raised Cos.
Heatmap width	
$\rightarrow$ as absolute margin width	$\{7, 19, 31\}$ px
$\rightarrow$ as std σ_{hm} of $h(\cdot)$	$\{1, 3, 5\}$ px

S9 Complementary figure and tables

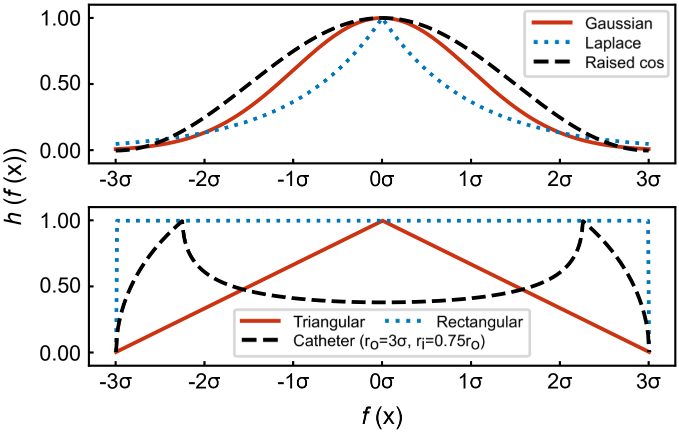

Fig. S9 Common choices for the distance-dependent function $h(\cdot)$.

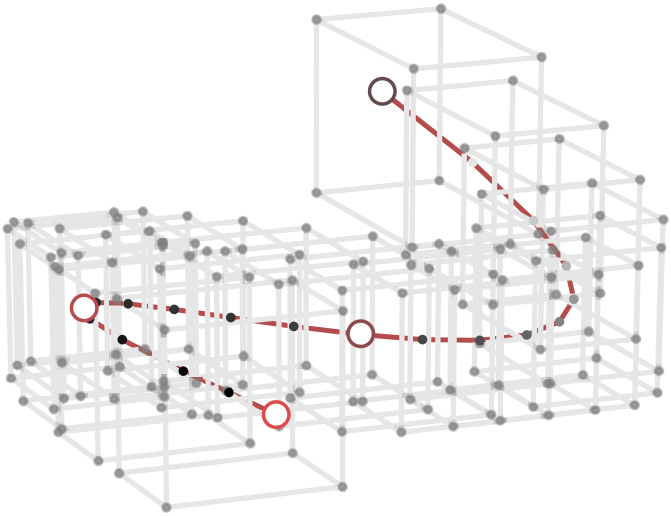

Fig. S10 Extension of localized distance evaluation to 3D. Each 3D bounding box is defined by four parallel line segments, which form a right rectangular prism of width w.

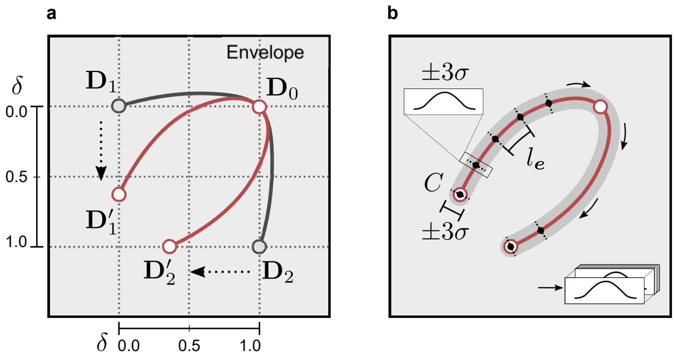

Fig. S11 Experiment and evaluation setup to estimate the approximation error of the proposed heatmap calculation. **a**: Curve configuration δ with step-wise narrowing of the curve legs. **b**: Evaluation of the heatmap approximation quality by sampling orthogonal profiles along the curve.

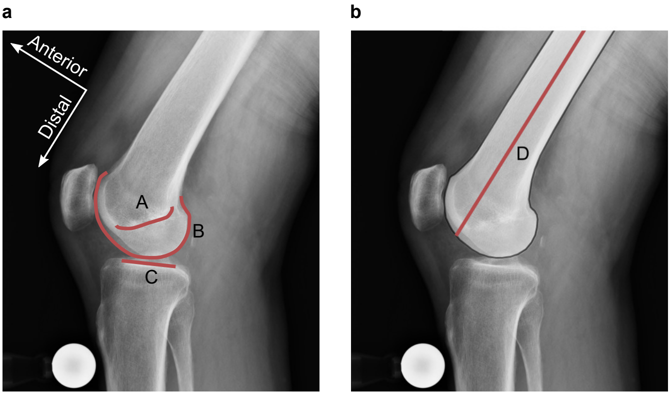

Fig. S12 Elongated anatomical features that are frequently used in operation planning for ligament reconstruction surgery. **a**, *direct* features which resemble salient anatomy. **b**, bone axis as *contextual* feature inferred from anatomical vicinity.

Table S9 ASSD [px] comparison for different methods for anatomical structure detection on knee joint radiographs.

**Method**	**Thr.**	**Blumensaat line**	**Femoral condyle**	**Tibial plateau**	**Femoral axis**
BCE	0.5	2.01 ± 1.84	0.72 ± 0.42	186.07 ± 31.47	145.37 ± 18.75
	Otsu	0.68 ± 0.24	$\mathbf{0.42 \pm 0.20}$	0.81 ± 0.44	1.14 ± 0.56
Dice	0.5	$\mathbf{0.65 \pm 0.29}$	0.60 ± 0.39	$\mathbf{0.78 \pm 0.52}$	3.26 ± 1.55
	Otsu	0.66 ± 0.30	0.60 ± 0.39	0.79 ± 0.58	3.26 ± 1.55
BCE+Dice [32]	0.5	1.05 ± 0.60	0.87 ± 0.82	0.87 ± 0.46	4.70 ± 3.84
	Otsu	1.04 ± 0.62	0.87 ± 0.82	0.87 ± 0.46	4.71 ± 3.84
W4BCE [33]	0.5	0.72 ± 0.26	0.54 ± 0.22	0.87 ± 0.47	1.24 ± 1.31
	Otsu	0.71 ± 0.23	0.54 ± 0.21	0.88 ± 0.48	1.36 ± 1.79
DeepFlux-P [26]					
context=16	0.4	1.06 ± 0.40	0.75 ± 0.19	0.87 ± 0.45	1.18 ± 0.56
Gauss. heatmap					
$\sigma_{\mathrm{HM}} = 1$ (close)	Otsu	0.66 ± 0.23	0.54 ± 0.25	0.84 ± 0.45	1.25 ± 0.68
$\sigma_{\mathrm{HM}} = 2$ (close)	Otsu	0.80 ± 0.26	0.59 ± 0.25	0.89 ± 0.44	$\mathbf{1.11 \pm 0.44}$
$\sigma_{\mathrm{HM}} = 3$ (close)	Otsu	0.82 ± 0.32	0.61 ± 0.20	0.87 ± 0.50	1.22 ± 0.70

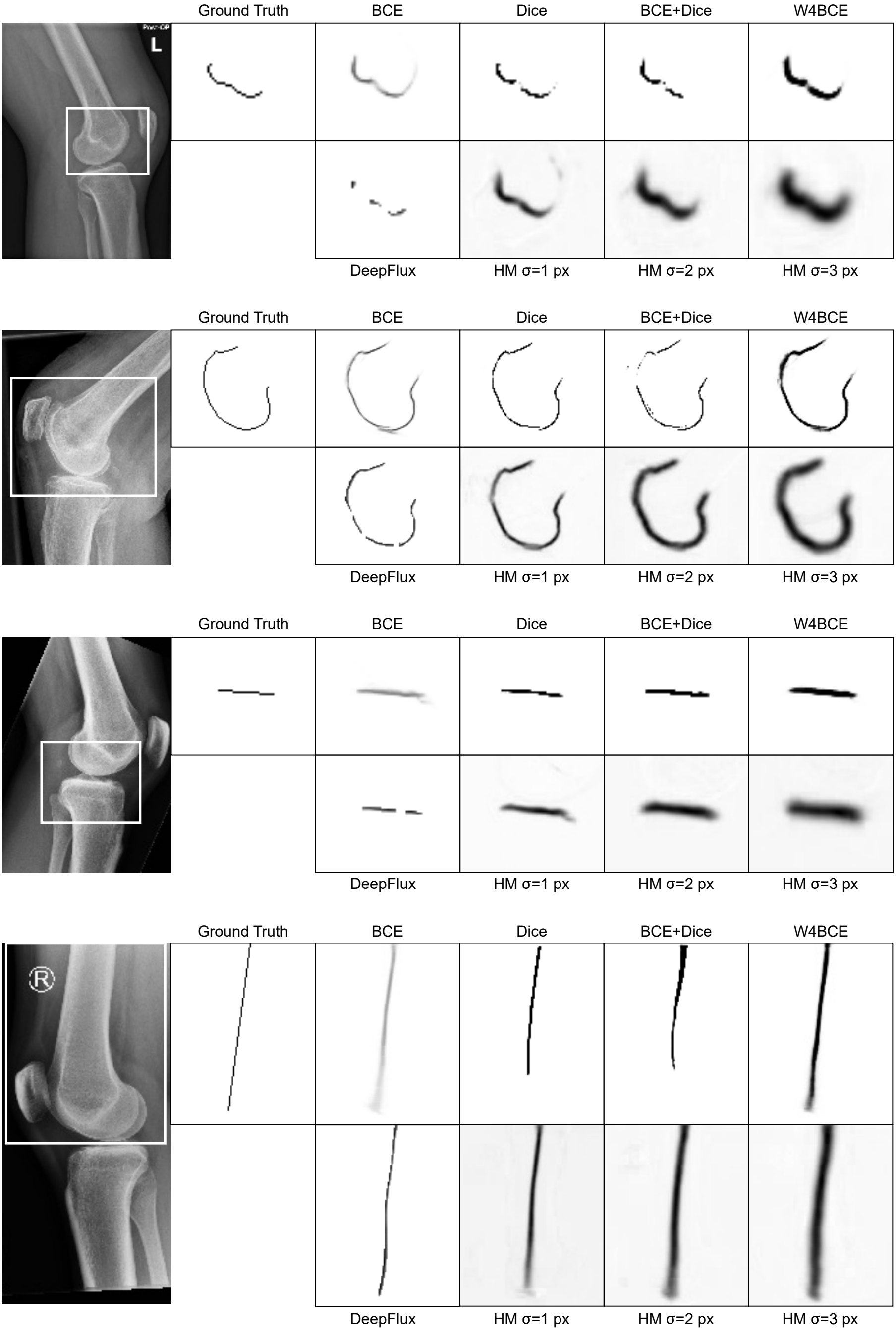

Fig. S13 Comparison of different methods for anatomical structure detection. The heatmap representation uses a close interpolation with no smoothing condition, i.e., $S = 0$.

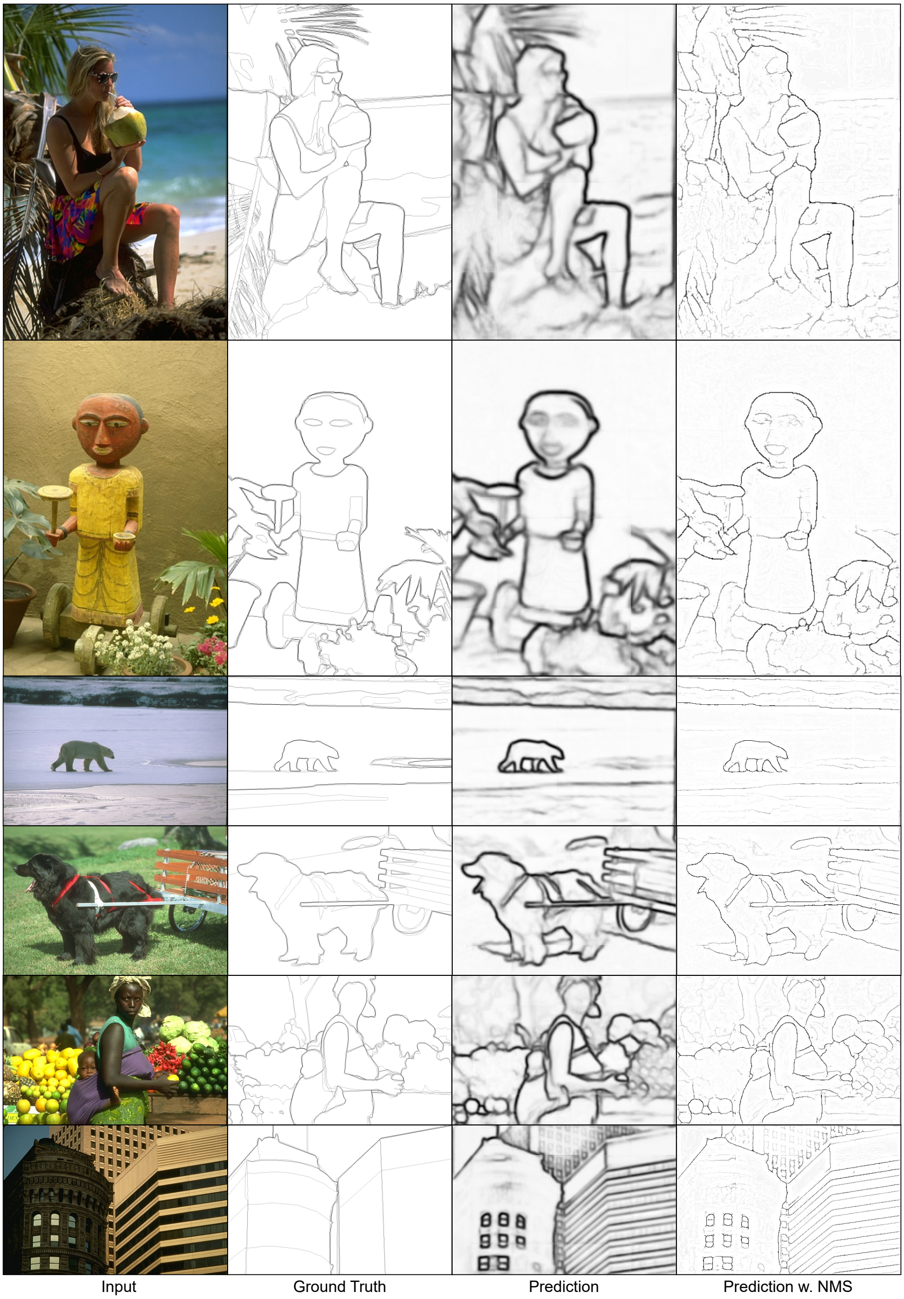

Fig. S14 Additional prediction images for the BSDS500 dataset [1].

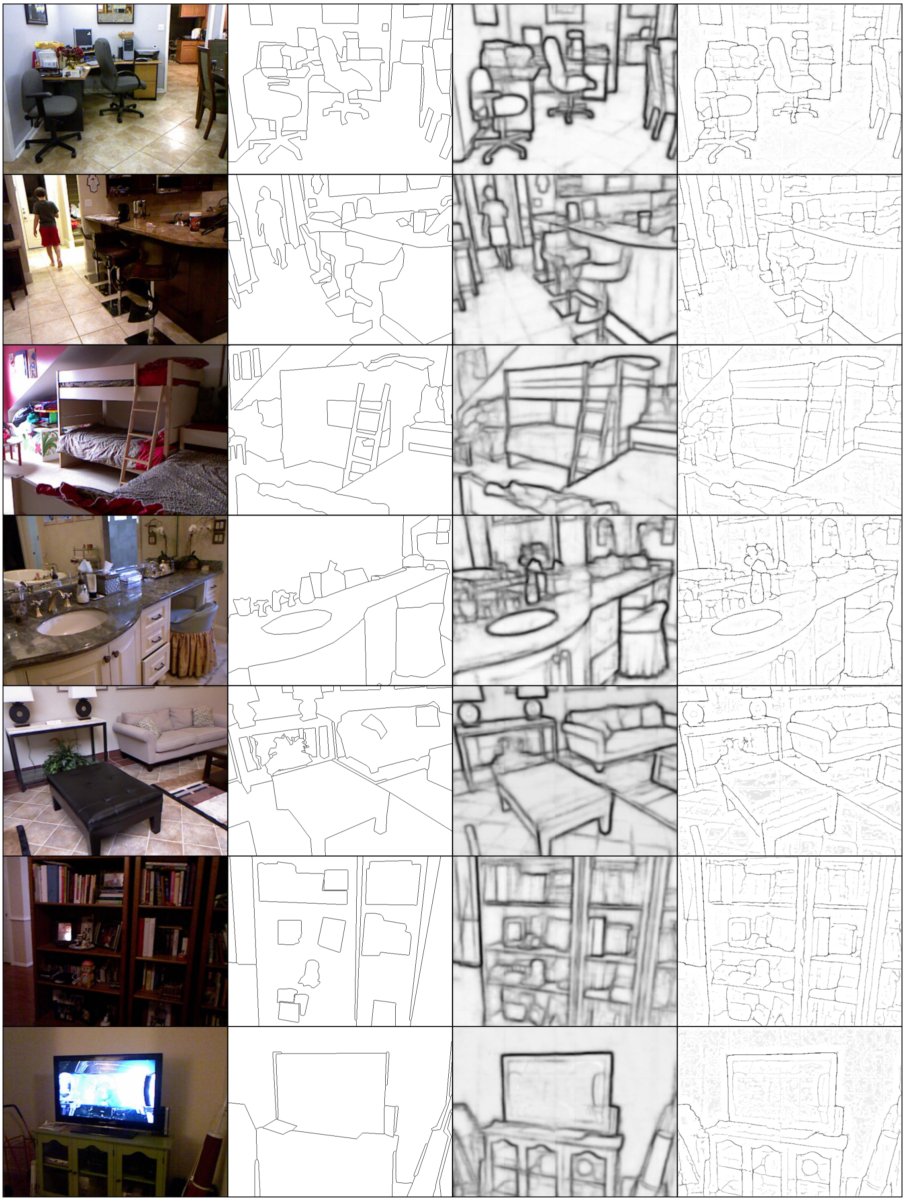

Fig. S15 Additional prediction images for the NYUD dataset [2].

References

[1] Arbelaez, P., Maire, M., Fowlkes, C., Malik, J.: Contour detection and hierarchical image segmentation. IEEE Trans. Pattern Anal. Mach. Intell. **33**(5), 898–916 (2011)

[2] Silberman, N., Hoiem, D., Kohli, P., Fergus, R.: Indoor segmentation and support inference from rgbd images. In: Fitzgibbon, A., Lazebnik, S., Perona, P., Sato, Y., Schmid, C. (eds.) Comput. Vision (ECCV)), pp. 746–760. Springer, Berlin, Heidelberg (2012)

[3] Shen, W., Bai, X., Hu, Z., Zhang, Z.: Multiple instance subspace learning via partial random projection tree for local reflection symmetry in natural images. Pattern Recognition **52**, 306–316 (2016)

[4] Shen, W., Zhao, K., Jiang, Y., Wang, Y., Zhang, Z., Bai, X.: Object skeleton extraction in natural images by fusing scale-associated deep side outputs. In: Conf. Comput. Vision Pattern Recognit. (CVPR), pp. 222–230 (2016)

[5] Shen, W., Zhao, K., Jiang, Y., Wang, Y., Bai, X., Yuille, A.: Deepskeleton: Learning multi-task scale-associated deep side outputs for object skeleton extraction in natural images. IEEE Trans. Imag. Processing **26**(11), 5298–5311 (2017). https://doi.org/10.1109/TIP.2017.2735182

[6] No author given: TuSimple Lane Detection Challenge. GitHub (2021)

[7] Demir, I., Koperski, K., Lindenbaum, D., Pang, G., Huang, J., Basu, S., Hughes, F., Tuia, D., Raskar, R.: Deepglobe 2018: A challenge to parse the earth through satellite images. In: Conf. Comput. Vision Pattern Recognit. (CVPR) (2018)

[8] Pu, M., Huang, Y., Guan, Q., Ling, H.: Rindnet: Edge detection for discontinuity in reflectance, illumination, normal and depth. In: IEEE Conf. Comput. Vision (ICCV), pp. 6879–6888 (2021)

[9] Liu, Y., *et al.*: Richer convolutional features for edge detection. Int. J. Comput. Vision **41**(8), 1939–1946 (2019). https://doi.org/10.1109/TPAMI.2018.2878849

[10] Sun, G., Yu, H., Jiang, X., Feng, M.: Adaptive feature pyramid network to predict crisp boundaries via nms layer and ods f-measure loss function. Information **13**(1) (2022). https://doi.org/10.3390/info13010032

[11] Zhou, Z., Siddiquee, M.M.R., Tajbakhsh, N., Liang, J.: Unet++: A nested u-net architecture for medical image segmentation. In: Deep Learning in Medical Image Analysis and Multimodal Learning for Clinical Decision

Support, pp. 3–11. Springer, Cham (2018)

[12] Xie, S., Tu, Z.: Holistically-nested edge detection. In: IEEE Conf. Comput. Vision (ICCV), pp. 1395–1403 (2015)

[13] Gupta, S., Arbelaez, P., Malik, J.: Perceptual organization and recognition of indoor scenes from rgb-d images. In: Conf. Comput. Vision Pattern Recognit. (CVPR), pp. 564–571 (2013)

[14] Gupta, S., Girshick, R., Arbeláez, P., Malik, J.: Learning rich features from rgb-d images for object detection and segmentation. In: Comput. Vision (ECCV), pp. 345–360 (2014). Springer

[15] Canny, J.: A computational approach to edge detection. IEEE Trans. Pattern Anal. Mach. Intell. **PAMI-8**(6), 679–698 (1986). https://doi.org/10.1109/TPAMI.1986.4767851

[16] Martin, D.R., Fowlkes, C.C., Malik, J.: Learning to detect natural image boundaries using local brightness, color, and texture cues. IEEE Trans. Pattern Anal. Mach. Intell. **26**(5), 530–549 (2004). https://doi.org/10.1109/TPAMI.2004.1273918

[17] Dollár, P., Zitnick, C.L.: Fast edge detection using structured forests. IEEE Trans. Pattern Anal. Mach. Intell. **37**(8), 1558–1570 (2014)

[18] Hallman, S., Fowlkes, C.C.: Oriented edge forests for boundary detection. In: Conf. Comput. Vision Pattern Recognit. (CVPR), pp. 1732–1740 (2015). https://doi.org/10.1109/CVPR.2015.7298782

[19] Shen, W., Wang, X., Wang, Y., Bai, X., Zhang, Z.: Deepcontour: A deep convolutional feature learned by positive-sharing loss for contour detection. In: Conf. Comput. Vision Pattern Recognit. (CVPR), pp. 3982–3991 (2015). https://doi.org/10.1109/CVPR.2015.7299024

[20] Bertasius, G., Shi, J., Torresani, L.: Deepedge: A multi-scale bifurcated deep network for top-down contour detection. In: Conf. Comput. Vision Pattern Recognit. (CVPR) (2015)

[21] Bertasius, G., Shi, J., Torresani, L.: High-for-low and low-for-high: Efficient boundary detection from deep object features and its applications to high-level vision. In: IEEE Conf. Comput. Vision (ICCV) (2015)

[22] Liu, Y., Cheng, M.-M., Fan, D.-P., Le Zhang, Bian, J.-W., Tao, D.: Semantic edge detection with diverse deep supervision. Int. J. Comput. Vision **130**(1), 179–198 (2022). https://doi.org/10.1007/s11263-021-01539-8

[23] Mottaghi, R., Chen, X., Liu, X., Cho, N.-G., Lee, S.-W., Fidler, S., Urtasun, R., Yuille, A.: The role of context for object detection and semantic segmentation in the wild. In: Conf. Comput. Vision Pattern Recognit. (CVPR) (2014)

[24] Liu, Y., Jiang, P.-T., Petrosyan, V., Li, S.-J., Bian, J., Zhang, L., Cheng, M.-M.: DEL: Deep Embedding Learning for Efficient Image Segmentation. In: Int. Joint Conf. Artif. Intell., pp. 864–870 (2018)

[25] Maninis, K.-K., Pont-Tuset, J., Arbeláez, P., Van Gool, L.: Convolutional oriented boundaries: From image segmentation to high-level tasks. IEEE Trans. Pattern Anal. Mach. Intell. **40**(4), 819–833 (2017)

[26] Xu, Y., Wang, Y., Tsogkas, S., Wan, J., Bai, X., Dickinson, S., Siddiqi, K.: Deepflux for skeleton detection in the wild. Int. J. Comput. Vision **129**(4), 1323–1339 (2021). https://doi.org/10.1007/s11263-021-01430-6

[27] Zhao, K., Shen, W., Gao, S., Li, D., Cheng, M.: Hi-Fi: Hierarchical feature integration for skeleton detection. In: Lang, J. (ed.) Int. Joint Conf. Artif. Intell., pp. 1191–1197 (2018). https://doi.org/10.24963/ijcai.2018/166

[28] Tsogkas, S., Kokkinos, I.: Learning-based symmetry detection in natural images. In: Fitzgibbon, A., Lazebnik, S., Perona, P., Sato, Y., Schmid, C. (eds.) Comput. Vision (ECCV), pp. 41–54. Springer, Berlin, Heidelberg (2012)

[29] Liu, Y., Cheng, M.-M., Hu, X., Wang, K., Bai, X.: Richer convolutional features for edge detection. In: Conf. Comput. Vision Pattern Recognit. (CVPR), pp. 3000–3009 (2017)

[30] Ke, W., Chen, J., Jiao, J., Zhao, G., Ye, Q.: SRN: Side-output residual network for object symmetry detection in the wild. In: Conf. Comput. Vision Pattern Recognit. (CVPR), pp. 1068–1076 (2017)

[31] Liu, C., Ke, W., Qin, F., Ye, Q.: Linear span network for object skeleton detection. In: Comput. Vision (ECCV), pp. 133–148 (2018)

[32] Deng, R., Shen, C., Liu, S., Wang, H., Liu, X.: Learning to predict crisp boundaries. In: Ferrari, V., Hebert, M., Sminchisescu, C., Weiss, Y. (eds.) Comput. Vision (ECCV) vol. 11210, pp. 570–586. Springer, Cham (2018). https://doi.org/10.1007/978-3-030-01231-1_35

[33] Holzmann, M., Davari, A., Seehaus, T., Braun, M., Maier, A., Christlein, V.: Glacier calving front segmentation using attention u-net. arXiv e-prints, 2101–03247 (2021) arXiv:2101.03247 [cs.LG]
